# Modulatory Effects of Phytochemicals on Gut–Brain Axis: Therapeutic Implication

**DOI:** 10.1016/j.cdnut.2024.103785

**Published:** 2024-05-22

**Authors:** Khojasteh Rahimi Jaberi, Vahab Alamdari-palangi, Amir Savardashtaki, Pooya Vatankhah, Tannaz Jamialahmadi, Amir Tajbakhsh, Amirhossein Sahebkar

**Affiliations:** 1 Department of Neuroscience, School of Advanced Medical Sciences and Technologies, Shiraz University of Medical Sciences, Shiraz, Iran; 2 Department of Molecular Medicine, School of Advanced Medical Sciences and Technologies, Shiraz University of Medical Sciences, Shiraz, Iran; 3 Department of Medical Biotechnology, School of Advanced Medical Sciences and Technologies, Shiraz University of Medical Sciences, Shiraz, Iran; 4 Infertility Research Center, Shiraz University of Medical Sciences, Shiraz, Iran; 5 Anesthesiology and Critical Care Research Center, Shiraz University of Medical Sciences, Shiraz, Iran; 6 Pharmaceutical Research Center, Pharmaceutical Technology Institute, Mashhad University of Medical Sciences, Mashhad, Iran; 7 Medical Toxicology Research Center, Mashhad University of Medical Sciences, Mashhad, Iran; 8 Pharmaceutical Sciences Research Center, Shiraz University of Medical Sciences, Shiraz, Iran; 9 Biotechnology Research Center, Pharmaceutical Technology Institute, Mashhad University of Medical Sciences, Mashhad, Iran; 10 Applied Biomedical Research Center, Mashhad University of Medical Sciences, Mashhad, Iran

**Keywords:** Centeral nervous system, medicinal plants, microbiota, neurodegeneration, polyphenol

## Abstract

This article explores the potential therapeutic implications of phytochemicals on the gut–brain axis (GBA), which serves as a communication network between the central nervous system and the enteric nervous system. Phytochemicals, which are compounds derived from plants, have been shown to interact with the gut microbiota, immune system, and neurotransmitter systems, thereby influencing brain function. Phytochemicals such as polyphenols, carotenoids, flavonoids, and terpenoids have been identified as having potential therapeutic implications for various neurological disorders. The GBA plays a critical role in the development and progression of various neurological disorders, including Parkinson’s disease, multiple sclerosis, depression, anxiety, and autism spectrum disorders. Dysbiosis, or an imbalance in gut microbiota composition, has been associated with a range of neurological disorders, suggesting that modulating the gut microbiota may have potential therapeutic implications for these conditions. Although these findings are promising, further research is needed to elucidate the optimal use of phytochemicals in neurological disorder treatment, as well as their potential interactions with other medications. The literature review search was conducted using predefined search terms such as phytochemicals, gut–brain axis, neurodegenerative, and Parkinson in PubMed, Embase, and the Cochrane library.

## Introduction

The central nervous system (CNS) and the enteric nervous system (ENS) communicate through the gut–brain axis (GBA), which involves a number of channels including hormonal, immunological, and neurological mechanisms [1]. It is essential for controlling many different physiological and psychological functions, such as hunger, digestion, mood, and thought processes [1]. Recent studies have shown that alterations in the gut microbiota and production of microbial metabolites are associated with a variety of immune-related neurological disorders, including epilepsy, Parkinson’s disease (PD), migraine, anxiety, depression, autism spectrum disorder (ASD), multiple sclerosis (MS), and neurodegenerative disorders [2,3]. Despite the importance of the gut microbiota for host health and disease states, the majority of prior research on this subject has only found correlations between particular clinical conditions and bacterial profiles [2]. However, data points to the possibility that some neurological disorders may be primarily caused by microbiome malfunction [2].

**FIGURE 1:**
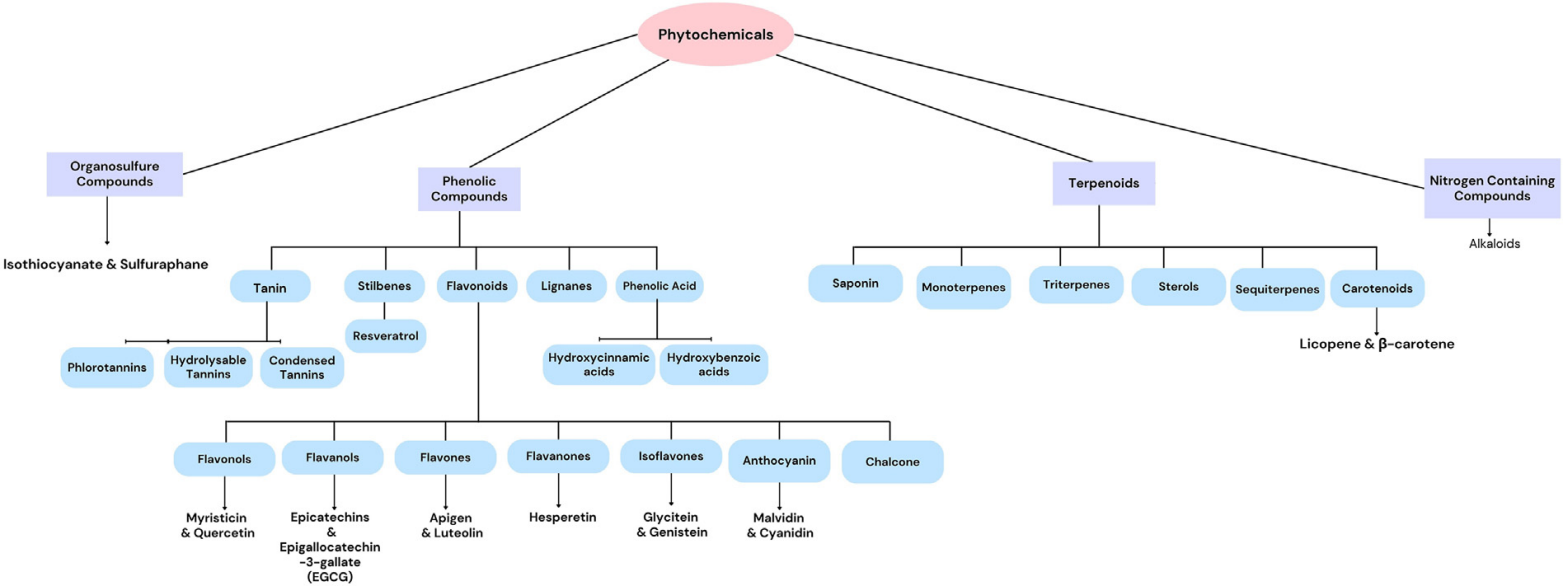
Modulatory effects of phytochemicals on the gut–brain axis. Abbreviations: Aβ, amyloid beta. BDNF, brain-derived neurotrophic factor; EGCG, epigallocatechin-3-gallate; GABA, γ-aminobutyric acid; HPA, hypothalamic-pituitary-adrenal; IL, interleukin; MPTP, 1-methyl-4-phenyl-1,2,3,6-tetrahydropyridine; MDA, malondialdehyde; NF-κB, nuclear factor kappa B; NLRP3, NLR family pyrin domain containing 3; TLR4, Toll-like receptor; TNF, tumor necrosis factor alpha; 6-OHDA, 6-hydroxydopamine.

Phytochemicals, which are compounds derived from plants, have been the subject of extensive research over the past few decades [[4], [5], [6], [7], [8], [9], [10], [11], [12], [13], [14], [15], [16], [17], [18]]. These natural products, which encompass different classes of compounds (Figure 1), have been demonstrated to exert regulatory effects on the GBA. These compounds have been shown to interact with the gut microbiota, immune system, and neurotransmitter systems, thereby influencing brain function. Phytochemicals such as polyphenols, carotenoids, flavonoids, and terpenoids have been identified as having potential therapeutic implications for various neurological disorders [19].

An increasing amount of evidence indicates that using phytochemicals to manipulate the GBA may be a promising treatment strategy for neurological conditions [20]. Phytochemicals have been demonstrated to impact the GBA in a variety of ways, including modulating gut microbiota composition, reducing inflammation, and improving neurotransmitter signaling, which may aid in delaying or preventing the onset of neurological illnesses and improve cognitive function in patients [20].

However, the translation of preclinical findings into clinical applications poses several challenges. One of the main challenges is optimizing the doses and delivery methods of phytochemicals. Many phytochemicals have poor bioavailability, meaning that they are difficult for the body to absorb and may not reach the brain in sufficient quantities to have therapeutic effects [21]. There is also a need to identify specific targets for phytochemicals in the brain to optimize their therapeutic effects [22].

It has been demonstrated that GBA is essential for the onset and course of a number of neurological conditions, such as PD, MS, depression, anxiety, and ASD [1]. The gut microbiota, composed of the gastrointestinal tract’s resident bacteria, is a crucial element of the GBA that may impact behavior and brain function through diverse pathways [19]. An imbalance in the makeup of the gut microbiota, or dysbiosis, has been linked to a number of neurological conditions, suggesting that modulating the gut microbiota may have potential therapeutic implications for these conditions [23]. Phytochemicals have been found to modulate the gut microbiota as well as to exert neuroprotective, anti-inflammatory, and antioxidant effects, which could be advantageous in the management and prevention of neurological conditions [24]. For example, curcumin, a polyphenol in turmeric, has been demonstrated to have anti-inflammatory, antioxidant, and gut microbiota-modulating qualities, all of which may be helpful in the treatment of MS [25]. Resveratrol is a polyphenol that may be found in grapes and red wine. Studies have indicated that it can modify the gut microbiota and have neuroprotective effects, which may help cure PD [3,26]. Numerous fruits and vegetables contain the flavonoid quercetin, which has been demonstrated to have anti-inflammatory and antioxidant qualities, as well as to modify the gut flora. These qualities may make quercetin useful in the treatment of depression [27].

However, further research is needed to elucidate the optimal use of phytochemicals in neurological disorder treatment, as well as their potential interactions with other medications. Here we discuss GBA, the variety of types of phytochemicals, the effects of phytochemicals in neurological disorders, especially Alzheimer’s disease (AD), therapeutic approaches, and limitations of the use of phytochemicals.

## Prevention and Treatment of Phytochemicals for GBA in Neurodegenerative Diseases (NDDs)

Comprehensive information about the GBA under physiological conditions, neurodegenerative diseases (NDDs)/brain disorders, and also an overview of phytochemicals with classification and bioavailability of phytochemicals are provided in Supplementary File 1.

In the last decade, researchers have shown that chemical components and metabolites affect health through the regulation of gut microbiota composition. Different studies have proven that the gut microbiota, sympathetic and parasympathetic nervous systems, ENS, CNS, and neuroimmune and neuroendocrine system pathways are correlated [28,29]. CNS homeostasis is vital for control of the relative constancy of the internal environment of an organism, and disruption of homeostasis preservation due to abnormal gut–brain communication leads to various neurodenerative diseases, such as neurodevelopmental disorders, neuropsychiatric conditions, PD, AD, and MS [30,31]. Many studies have shown that regular consumption of phytochemicals reduces the risk of several neurological diseases by influencing the gut microbiota [32,33]. Therefore, modulating the GBA through active compounds of plant-based functional foods (phytochemicals) is a promising approach for preventing or treating mental health disorders, including Huntington’s disease, PD, and AD.

### Polyphenolic compounds

Polyphenolic compounds (such as curcumin, stilbenes, lignans, flavonoids, lignins, benzoic acid, cinnamic acid, and coumarins) found in our diet can affect the gut microbiota, leading to the production of polyphenolic compounds that have therapeutic benefits and can better permeate the blood–brain barrier (BBB) [34,35]. The effectiveness of polyphenols as beneficial antioxidants has been questioned due to conflicting research on their bioavailability [36]. However, recent studies suggest that polyphenols can still have biological effects through chemical modifications carried out by the gut microbiota [37,38]. Enzymes in the gut microbiota can modify polyphenols by removing sugar molecules, adding hydroxyl groups, and removing methyl groups, resulting in smaller breakdown products that are easily absorbed in the intestines [39,40]. These breakdown products can be divided into 2 categories: some have even higher biological activity than the original compound, while others lose their biological activity. This suggests that targeting the GBA could be a promising approach to treating serious neurological disorders.

### Curcumin

Curcumin is a naturally occurring compound that belongs to a class of chemicals called polyphenols that exhibits various biological activities such anti-inflammatory, antioxidant, and anticancer properties [14,[41], [42], [43], [44], [45], [46], [47], [48]]. These properties have led researchers to explore its potential therapeutic applications, especially in the management of neurodegenerative and neurological diseases [49,50]. Curcumin’s pharmacological benefits are limited due to its low water solubility, instability in chemical composition, quick metabolism, and poor bioavailability [51]. One hypothesis that could explain how curcumin has a neuroprotective effect despite its limited availability is that it indirectly affects the CNS by influencing the “microbiota-GBA.” This 2-way system axis plays an important role in maintaining brain health. Curcumin is modified by bacterial enzymes, resulting in metabolites that are more pharmacologically active than curcumin itself. Curcumin and its metabolites may help restore imbalances in the gut microbiome [51,52]. Curcumin is transformed not only by enzymes in the body but also by those produced by gut microbiota. Various microorganisms are capable of modifying curcumin, and the composition of an individual’s microbiota determines the biotransformation of dietary curcumin. Different bacterial strains, such as Bifidobacteria and Lactobacilli, produce different curcumin metabolites through various metabolic processes, including hydroxylation, demethylation, reduction, and demethoxylation [53,54]. Rajeswari and Sabesan [55] conducted a study on the effects of curcumin and tetrahydrocurcumin (ThC) on PD in mice. The disease was induced by 1-methyl-4-phenyl-1,2,3,6-tetrahydropyridine (MPTP), which decreased dopamine (DA) and 3,4-dihydroxyphenylacetic acid (DOPAC) levels while increasing monoamine oxidase (MAO-B) activity. When curcumin (80 mg/kg intraperitoneal [i.p.]) and ThC (60 mg/kg i.p.) were administered systemically, they significantly reversed the depletion of DA and DOPAC caused by MPTP, as well as inhibited MAO-B activity [55]. Gao et al. [56] demonstrated that intraperitoneal injection of ThC, an active metabolite of curcumin, increased the expression of autophagy-associated proteins LC3-II and Beclin-1 24 h after traumatic brain injury (TBI). ThC treatment also reduced the expression of malondialdehyde (MDA) and increased glutathione peroxidase activity. Additionally, ThC treatment mitigated apoptosis by regulating mitochondrial apoptosis and reducing oxidative stress. The activation of autophagy was hindered and the inhibitory effect of ThC on the translocation of Bax to the mitochondrial membrane was reversed by treatment with 3-methyladenine. Furthermore, ThC treatment improved neurological function and decreased brain water content in rats after TBI [56]. Curcumin administration in mice with AD improved spatial learning and memory abilities, reduced amyloid plaques in the hippocampus, and altered the composition of bacterial taxa, such as Lactobacillaceae*,* Rikenellaceae, Bacteroidaceae, Bacteroides*,* Prevotellaceae*,* Parabacteroides, and Prevotella, which are associated with AD [57].

### Flavan-3-ols

The flavan-3-ols are metabolized by gut bacteria, producing several aryl-γ-valerolactone and arylvaleric acid derivatives. These derivatives were identified as the primary compounds that provide protection against AD, as shown in mouse AD models [58]. Valerolactones and their metabolites have been found to selectively eliminate amyloid beta (Aβ) oligomers, protecting against memory loss in mouse models of AD. Furthermore, the breakdown of valerolactones results in the formation of phenolic or polyphenolic degradation products, including (hydroxyaryl)cinnamic acid, (hydroxyaryl)valeric acid, (hydroxyaryl)acetic acid, (hydroxyaryl)propanoic acid, and derivatives of hydroxybenzoic acid. These secondary metabolites are easier for the body to absorb, can pass through the BBB more easily than the flavonoids found in food, and can help reduce inflammation in the brain [59]. Through a series of experiments, including computer analysis and in vitro and in vivo studies, researchers identified metabolites with a high potential to pass through the BBB. In vivo studies conducted on rats injected with pure 5-(3′,4′-dihydroxyphenyl)-γ-valerolactone confirmed the presence of 5-(hydroxyphenyl)-γ-valerolactone-sulfate (3′,4′ isomer) in the brain. This research demonstrated the BBB permeability of one of the main trifluorooxonium-derived metabolites using different experimental models, which may contribute to understanding the potential neuroprotective effects of phenolic-rich foods in the context of the GBA [60].

### Ellagitannins (ETs)

Ellagitannins (ETs) are a type of polyphenols found in various fruits and nuts, such as pomegranates, raspberries, and walnuts. Recent studies have shown that ETs have potential health benefits, including neuroprotective, antioxidant, anticancer, and anti-inflammatory properties [61,62]. Despite the various biological benefits of ETs, their limited bioavailability makes it difficult to achieve significant concentrations in the body. In contrast, urolithins (6H-dibenzo[b,d]pyran-6-one derivatives), which are metabolites of ETs produced by gut microbiota, are more easily absorbed and may be the bioactive compounds responsible for the observed benefits of ETs, such as neuroprotective, anti-inflammatory, and antioxidant effects [63].

### Urolithins and pomegranate (*Punica granatum*) extract

The use of in silico computational studies to predict BBB permeability revealed that only urolithins, and not any of the other constituents of pomegranate (*Punica granatum*) extract, met the necessary criteria for penetration. In vitro studies showed that urolithins were able to prevent the fibrillation of Aβ, while methyl-urolithin B had a protective effect in *Caenorhabditis elegans* following induction of Aβ-induced neurotoxicity and paralysis. In contrast, neither *Punica granatum* extract nor its predominant ETs had a protective effect. These findings suggest that urolithins are the compounds in pomegranate that are able to cross the BBB and contribute to its anti-AD effects [63]. Xu et al. [64] demonstrated that urolithins A and B can lower levels of nitric oxide and reduce the expression of proinflammatory genes (TNF-α, IL-6, IL-1β, inducible nitric oxide synthase [iNOS], and cyclooxygenase-2 [COX-2]) in microglia treated with lipopolysaccharides (LPS). In addition, urolithins A and B can inhibit the activation of signaling pathways (ERK1/2, p38 MAPK, Akt, nuclear factor kappa B [NF-κB]) involved in inflammation [64].

Silibinin is a flavonoid compound that has been used to protect the liver and brain in the clinical treatment of liver and brain diseases. It significantly reduced memory damage caused by LPS treatment in rats by decreasing the level of IL-1β and increasing the level of IL-4 in the hippocampus, attenuated NF-κB expression, and increased the generation of total reactive oxygen species (ROS) in the hippocampus as well as the expressions of brain-derived neurotrophic factor (BDNF) and tyrosine receptor kinase B (TrkB). Silibinin also reversed the LPS-induced reduction of neurons in the hippocampus. These results suggest that silibinin can improve learning and memory impairment caused by LPS by activating the ROS–BDNF–TrkB pathway in the hippocampus and suppressing the inflammatory response [65].

Tryptophan is involved in many physiological and pathological processes in the body. It can be absorbed in the small intestine and transported to other parts of the body, converted to serotonin, or broken down into other metabolites [66]. Tryptophan can also be metabolized by gut bacteria, which can affect inflammation in the body [67]. Serotonin, which is produced from tryptophan, is an important neurotransmitter that plays a role in emotion processing, learning, and memory [68]. Tryptophan is also involved in neurodevelopment and can influence the natural history of diseases, such as inflammatory bowel disease, neurodegenerative disorders, neurodevelopmental disorders, and cerebrovascular disorders [69].

Several studies in animal models have demonstrated that flavonoids reduce oxidative stress, decrease neuroinflammation, stimulate neurogenesis, activate neuronal regeneration, and protect the nervous system through several various mechanisms [70,71]. Some specific flavonoids have the ability to cross the BBB and provide direct neuroprotective effects by inhibiting oxidative stress, reducing inflammatory responses, regulating neuronal metabolism, and promoting neuronal regeneration [72]. Additionally, some studies suggest that flavonoids can indirectly protect the nervous system by modulating the composition and metabolites of gut microbiota that have an impact on the function of the GBA [73,74].

The indirect effects of flavonoids, such as their ability to modulate gut microbiota and the GBA, may have a more significant impact than their direct effects on the CNS [72]. Research has shown that flavonoids have the ability to control the growth of specific bacterial groups and modify the structure and function of gut microbiota [75]. Flavonoids have the potential to inhibit the growth and colonization of potentially harmful bacterial groups, such as *Escherichia coli* and *Staphylococcus aureus*, in the gut [76]. Furthermore, flavonoids act as metabolic substrates for beneficial bacteria, such as Bifidobacterium and Lactobacillus species, which promote their growth and proliferation [76]. This ensures a stable and beneficial gut community that is significant for the health of not only the gut but also other organs, such as the brain.

Flavonoids can also promote the production of different metabolites, including short-chain fatty acids (SCFAs), γ-aminobutyric acid, and BDNF. Some of these metabolites may be converted into neurotransmitters through biological processes [77]. 8-Dihydroxyflavone is a small-molecule TrkB agonist that has shown promising results in reversing memory deficits and β-site amyloid precursor protein (APP) cleaving enzyme 1 (BACE1) elevation in a mouse model of AD [78]. Recently, it was shown that 7,8-dihydroxyflavone (7,8-DHF) can cross the BBB and bind to the TrkB receptor, which is involved in neuronal survival, differentiation, and synaptic plasticity [79]. Activation of TrkB signaling has been shown to promote the growth and survival of neurons and to enhance synaptic plasticity, which is important for learning and memory [80]. Devi and Ohno [78] investigated the impact of 7,8-DHF in the 5XFAD transgenic mouse model of AD. 5XFAD mice and nontransgenic littermate controls were given 7,8-DHF (5 mg/kg, i.p.) once daily for 10 consecutive days when they were 12 to 15 mo old. Devi and Ohno discovered that 7,8-DHF improved the memory deficits of 5XFAD mice in the spontaneous alternation Y-maze task. The hippocampal BDNF–TrkB pathway was impaired in 5XFAD mice, as shown by significant reductions in BDNF, TrkB receptors, and phosphorylated TrkB. 7,8-DHF restored deficient TrkB signaling in 5XFAD mice without affecting endogenous BDNF levels. In addition, 5XFAD mice had increased levels of BACE1, which initiates Aβ generation, similar to sporadic AD. 7,8-DHF prevented BACE1 elevation and reduced the levels of the β-secretase-cleaved C-terminal fragment of APP, Aβ40, and Aβ42 in the brains of 5XFAD mice. Furthermore, 7,8-DHF reduced BACE1 expression in wild-type mice, indicating that BDNF–TrkB signaling is also important for regulating baseline levels of BACE1. Their findings suggest that systemic administration of 7,8-DHF can improve AD-associated memory deficits by reducing BACE1 expression and β-amyloidogenesis [78].

The severity of AD is closely related to the loss of synapses in the brain. The synaptic dysfunction in AD is caused by a deficiency in the signaling pathway of BDNF and TrkB [81]. Zhang et al. [82] investigated the impact of 7,8-DHF on neurotoxicity and synaptogenesis caused by Aβ in vivo. They administered 7,8-DHF orally to the 5XFAD transgenic mouse model of AD, which has 5 mutations related to familial AD. The treatment began before plaque deposition at 2 mo of age, and the mice were evaluated for cognitive performance and AD-like neuropathology at 6 mo of age. The study found that 7,8-DHF protected primary cortical neurons and locus coeruleus neurons from Aβ-induced toxicity, promoted dendritic growth and synaptogenesis, and prevented Aβ deposition, hippocampal synapse loss, synaptic dysfunction, and spatial memory deficits in 5XFAD mice [82].

### Apigenin

Apigenin (4′,5,7-trihydroxyflavone), a major plant flavone, is a pharmacologically active agent that possesses anticancer, anti-inflammatory, and antioxidant properties and is used to treat various human diseases [83]. Zhao et al. [84] investigated the impact of apigenin on cognitive function in mice with AD. Their study found that 3 mo of oral treatment with apigenin improved learning deficits and memory retention in these mice. Apigenin also had positive effects on APP processing, reducing the accumulation of Aβ plaques by downregulating BACE1 and β-C-terminal fragment levels. Additionally, apigenin exhibited antioxidant properties by scavenging superoxide anions and enhancing the activity of antioxidative enzymes. It also restored the neurotrophic ERK/CREB/BDNF pathway in the cerebral cortex. Their findings suggest that apigenin has the potential to alleviate AD-related cognitive impairment by reducing Aβ burden, inhibiting amyloidogenic processes, mitigating oxidative stress, and restoring the ERK/CREB/BDNF pathway [84]. Quercetin is a flavonoid that possesses antioxidant and anti-inflammatory properties [85]. It can also cross the BBB and has a neuroprotective effect by increasing the resistance of neurons to oxidative stress and excitotoxicity [86]. However, its low oral bioavailability limits its clinical use. To address this, researchers evaluated the potential of nanoencapsulated quercetin in zein nanoparticles (NPQ) as an oral treatment of AD. SAMP8 mice were treated with either NPQ or a quercetin solution for 2 mo. The results showed that NPQ significantly improved cognition and memory impairments in the mice and decreased the expression of the hippocampal astrocyte marker glial fibrillary acidic protein [87].

### Quercetin-3-*O*-glucuronide (Q3G)

Quercetin-3-*O*-glucuronide (Q3G), a metabolite of quercetin, can protect the brain in AD by reducing Aβ accumulation and tau phosphorylation and improves cognitive function in AD-like mice. Q3G can also help restore gut microbiota dysbiosis caused by Aβ. It increases the abundance of g_Alistipes and g_Rikenella and decreases g_Barnesiella and g_Lactobacillus in the Aβ group, which correlates with inflammatory factors in the brain. Q3G treatment can restore the abundance of these gut microbiota to normal levels, preventing neuroinflammation. Additionally, Q3G can help restore the reduction in SCFAs caused by Aβ42, which is related to changes in gut microbiota [88]. When only starch is available as an energy source, the gut bacterium *Eubacterium ramulus* relies on interactions with other bacterial species to metabolize quercetin, a commonly consumed flavonoid. *E. ramulus* can degrade quercetin in the presence of glucose, but not when starch is the sole energy source. However, the presence of *Bacteroides thetaiotaomicron*, a starch-metabolizing bacterium that does not metabolize quercetin, stimulates the degradation of quercetin and the production of butyrate by *E. ramulus* through cross-feeding of glucose and maltose molecules released from starch [89]. Sodium butyrate had neuroprotective effects in PD by improving cognitive behavior and coordination, preventing dopaminergic degeneration and cell death in the brain, upregulating proteins associated with the BBB, increasing the expression of Bcl-2, decreasing the expression of Bax, and increasing the levels of colonic glucagon like peptide-1 (GLP-1) and cerebral GLP-1 receptor expression [90]. Long-term treatment with the histone deacetylase inhibitor sodium butyrate improved associative memory in an AD mice model (APPPS1-21), even at an advanced stage of pathology. The improvement in memory was associated with increased histone acetylation in the hippocampus and enhanced expression of genes related to associative learning [91]. In addition, sodium butyrate had effects on reducing Aβ levels in the brain and improving associative learning and cognitive function [92].

### Isoorientin

Isoorientin (or homoorientin) is a flavone, a chemical flavonoid-like compound, that can help treat NDDs by regulating gut microbiota. It reduces Aβ plaque deposition, decreases the levels of TNF-α, IL-6, iNOS, and COX-2, and increases the levels of IL-4 and IL-10 in AD mice. Additionally, it promotes the growth of specific microbiota in the fecal and cecal microbiota of AD mice [29]. Ali et al. [93] investigated the effectiveness of anthocyanin-loaded PEG-AuNPs in enhancing the neuroprotective efficacy of anthocyanins in an Aβ1–42 mouse model of AD. They found that both treatments improved memory impairments, but the anthocyanin-loaded PEG-AuNPs were more effective. The study also showed that the anthocyanin-loaded PEG-AuNPs protected pre- and postsynaptic proteins, regulated the p-PI3K/p-Akt/p-GSK3β pathway, and prevented hyperphosphorylation of tau protein, inhibiting apoptosis and neurodegeneration in the Aβ1–42-injected mice. This effect was similar to the outcomes observed with the use of quercetin nanoparticles [93].

### Neurotrophins

Neurotrophins are essential for the survival, maintenance, and regeneration of specific neurons in the brain. Prominent neurotrophins are nerve growth factor (NGF), BDNF, NT-3, and NT-4/5 [94]. Reduced levels of neurotrophins are linked to NDDs, and NGF is widely studied as a drug target for these conditions [95]. Other potential targets include antioxidants, anti-inflammatory agents, antistress factors, and acetylcholinesterase inhibitors [96]. Neurotrophins hold promise for developing neuroprotective agents, and administering them may be a viable treatment of NDDs. Although clinical trials pose challenges, phytochemicals and synthetic derivatives have shown potential in regulating neurotrophin levels. Modulators or enhancers that target the Trk receptor could be valuable in restoring neurotrophin levels [97]. Some neurotrophins cannot penetrate the BBB, but this can be addressed by using neurotrophin-mimetic compounds or compounds that stimulate neurotrophin expression and can cross the BBB.

### (-)-Epigallocatechin-3-gallate (EGCG)

(-)-Epigallocatechin-3-gallate (EGCG) is a polyphenolic compound found in green tea, which has been reported to have various health benefits. A recent study suggested that EGCG may also have a positive effect on learning and memory deficits in AD model mice [98]. In a study conducted by Liu et al. [99], it was discovered that administering EGCG treatment (2 mg/kg/d) improved cognitive impairments, reduced the overexpression of Aβ(1–40) and APP, and prevented neuronal apoptosis in mice with APP/PS1. It was also noted that EGCG treatment increased the expression of NGF by raising the NGF/proNGF ratio in the same mice. Additionally, TrkA signaling was activated by EGCG treatment, which led to the phosphorylation of TrkA, c-Raf, ERK1/2, and CREB. At the same time, p75NTR signaling was significantly inhibited by reducing the expression of p75ICD, JNK2 phosphorylation, and cleaved-caspase 3 expression. As a result, Aβ deposits and neuronal apoptosis in the hippocampus were prevented [99]. In a study that investigated the therapeutic effect of curcumin on hippocampal damage in a rat model of PD, the results indicated that curcumin administration increased body weight, reversed anhedonia, and ameliorated behavioral manifestations in PD rats. Curcumin also increased the contents of DA and norepinephrine in hippocampal homogenates and alleviated 6-hydroxydopamine (6-OHDA)-induced hippocampal damage. Additionally, curcumin upregulated BDNF, TrkB, and PI3K protein expressions in the hippocampus, suggesting that curcumin may mediate neuroprotection by activating the BDNF/TrkB-dependent pathway to promote neural regeneration of hippocampal tissue [100]. Carito et al. [101] administered olive polyphenols to mice for 15 d. The olive polyphenols decreased glutathione levels and increased NGF and BDNF levels in the serum. In the brain, NGF and BDNF levels decreased in the hippocampus and striatum but increased in the olfactory lobes and hypothalamus. Their study suggests that olive polyphenols can activate the olfactory system by increasing NGF and BDNF levels but may also induce stress by affecting NGF/BDNF levels in the hippocampus and serum glutathione levels [101].

It has been proven that NDDs, such as AD and PD, are linked to oxidative damage, mitochondrial dysfunction, and neuroinflammation [102]. Phytochemicals, including curcumin, propolis, resveratrol, ginsenosides, and PUFAs, have anti-inflammatory properties that can modulate and suppress neuroinflammation through various approaches [103]. These phytochemicals can decrease neuroinflammation in the brain through several methods, including reducing systemic inflammation via the BBB, directly entering the brain to provide neuroprotection, improving the integrity of the disrupted BBB, and signaling to the brain through vagal reflex-mediated nutrition and protection from gastrointestinal function [104].

### Ginsenosides Rg1

Ginsenosides Rg1, an active component of ginseng, has the potential to be used as a therapeutic for PD by protecting dopaminergic neurons and reducing aberrant α-synuclein-mediated neuroinflammation. Oral treatment with ginsenoside Rg1 significantly reduced MPTP-induced mortality, behavior defects, loss of DA neurons, and abnormal ultrastructure changes in the substantia nigra pars compacta (SNpc). The protective effect of Rg1 may be due to its antineuroinflammatory properties. Rg1 regulated MPTP-induced reactive astrocytes and microglia, decreased the release of cytokines such as TNF-α and IL-1β in the SNpc, and alleviated the unusual MPTP-induced increase in oligomeric, phosphorylated, and disease-related α-synuclein in the SNpc [105].

### Resveratrol

Resveratrol, a natural polyphenol, possesses antiaging and anti-inflammatory characteristics that can help counteract the effects of stress [[106], [107], [108], [109], [132]]. Studies have shown that resveratrol has beneficial effects on a range of metabolic and CNS ailments, including diabetes, obesity, dementia, and depression [110,111], although controversial findings in clinical studies also exist [[112], [113], [114], [115]]. Additionally, it has been suggested that resveratrol possesses antiaging properties and can regulate inflammation in different parts of the body [116]. Resveratrol can impact the GBA in 3 ways: regulating gut and brain balance through the GLP-1 pathway, affecting gut microbiota diversity, and contributing to the balance between gut and brain function through the 5-hydroxytryptamine (5-HT) system [117]. Resveratrol administration before chronic-acute combined stress improved depression and anxiety-like behaviors and altered intestinal motility and visceral hypersensitivity in a rat model of irritable bowel syndrome (IBS). These improvements were attributed to the differential regulation of 5-HT levels in the brain and intestine. However, the effects of resveratrol were blocked by the 5-HT1A receptor antagonist NAN-190 hydrobromide, suggesting that 5-HT1A-related signaling is important in treating GBA dysfunction in IBS-like animal models [118]. Resveratrol balances Th1/Th2 toward Th2 polarization and shifts T_reg_/Th17 balance toward T_reg_ in the small intestinal lamina propria, reduces proinflammatory cytokine expression, and attenuates cerebral ischemia-induced increase in the permeability of the small intestine’s epithelial and vascular layers. It also protects against poststroke inflammation-induced BBB disruption and results in smaller cerebral infarcts and fewer neurological deficits [119]. Various preclinical and clinical studies have demonstrated the potential of phytochemicals for prevention and treatment of neurodegenerative disorders, such as PD and AD via the GBA (Table 1).

**TABLE 1:** Phytochemical effect on neurodegenerative disease via the gut–brain axis

Phytochemical classification	Bioactive compound	Dose	Disease	Model	Effects on NDD
Phenolic (flavonoid) [28]	Phenyl-γ-valerolactones	—	AD	Mouse model	Reduced memory deterioration as well as neuroinflammation in a mouse model of Aβ oligomer-induced memory impairment.
Phenolic (flavonoid) [29]	Isoorientin	25, 50 mg/kg	AD	Mouse model	Isoorientin treatment decreased Aβ42-positive deposition in the cortex and hippocampus.
Phenolic (flavonoid) [88]	Quercetin-3-O-glucuronide	—	AD	Mice and SH-SY5Y Cells	Quercetin-3-O-glucuronide alleviated brain insulin resistance by either directly targeting the brain or affecting the communication between the gut and brain. The treatment aims to alleviate cognitive dysfunction caused by Aβ1-42.
Phenolic (flavonoid) [120]	Quercetin	—	PD	Rat model	Quercetin improved neurochemical parameters, indicating the advantages of both symptomatic and neuroprotective treatments.
Phenolic (flavonoid) [121]	Quercetin	50 mg/kg	Repeated mild traumatic brain injury	Mouse model	Quercetin improved the neuropsychiatric issues via remodeling of the microbiome gut–brain axis.
Phenolic (flavonoid) [122]	Fisetin	100 ng/kg body weight	PD	Mouse model	Fisetin exerted a neuroprotective effect on neurodegeneration by altering the composition and diversity of gut microbiota.
Phenolic (flavonoid) [123]	Curcumin	25, 100, 400 mg/kg	PD	Mouse model	Curcumin exerted a protective effect on the progression of PD by modulating the gut microbiota-metabolite axis. Aerococcaceae and Lactobacillaceae, along with key metabolites, (e.g., dopa and tyrosine) play a dominant role in Curcumin-associated neuroprotection.
Phenolic (flavonoid) [57]	Curcumin	50, 200 mg/kg	AD	Mouse model	Curcumin altered bacterial species associated with AD development.
Phenolic (stilbenes) [124]	Resveratrol	—	AD	Mouse model	Resveratrol-selenium-peptide nanocomposites improves cognitive disorder by effectively inhibiting Aβ deposition in the hippocampus, downregulating Aβ-induced neuroinflammation, and alleviating gut microbiota disorder-related bacteria, such as Faecalibaculum, Rikenella, Alistipes, and Helicobacter.
Terpenoid (carotenoid) [110]	Fucoxanthin	—	AD	Aβ oligomer-injected mice	Fucoxanthin reduced the formation of Aβ fibrils and oligomers and attenuated cognitive impairment.
Phenolic (flavonoid) [125]	Equol	10, 20 μM	PD	SH-SY5Y cells	Equol exerted neuroprotective effects by decreasing 6-OHDA and MPP^+^-induced cytotoxicity.
Terpenoid (carotenoid) [104]	Lycopene	—	AD	Rat model	Lycopene improved attenuation of inflammatory injury and cognitive deficits by blocking the activation of NF-κB p65 and TLR4 expression.
Phenolic (flavonoid) [126]	Hesperidin	50 mg/kg	Mild traumatic brain injury	Mouse model	Hesperidin reduced depression-related symptoms in mTBI-induced mice by decreasing IL-1β, TNF-α, and MDA levels and increasing BDNF levels.
Phenolic (phenolic acid) [127]	Ferulic acid	20, 40, 80 mg/kg	Chronic unpredictable mild stress	Mouse model	Ferulic acid increased sucrose preference and decreased immobility time in mice by decreasing NLRP3 inflammasomes and inhibiting microglia activation.
Phenolic (flavonoid) [128]	Naringin	20, 40, 80 mg/kg	HD	Rat model	Naringin protected the nervous system from QA-induced damage by regulating oxidative and nitrosative stress, neuroinflammation, apoptosis, and mitochondrial complex activity.
Phenolic (tannin) [129]	Urolithin A	1–10 μM	AD	SH-SY5Y-APP695 cells	Urolithin A had neuroprotective effects by inducing transcription of several genes related to mitochondrial biogenesis.
Phenolic (tannin) [29]	Urolithin A	20 mg/kg	PD	BV2 microglial cells and mouse model	Urolithin A reduced the loss of dopaminergic neurons, and ameliorated behavioral deficits and neuroinflammation.
Terpenoid (carotenoid) [130]	Astaxanthin	—	Spinal cord injury	Rat model	Astaxanthin decreased the expression of inflammatory signaling mediators and cytokines following compression spinal cord injury.
(Phenolic) phenolic acid [131]	Gallic acid	100 mg/kg	PD	Rat model	Gallic acid improved symptoms of PD induced by rotenone.

Abbreviations: Aβ, amyloid-β; AD, Alzheimer’s disease; BDNF, brain-derived neurotrophic factor; HD, Huntington’s disease; IL-1β, interleukin-1 beta; NDD, neurodegenerative disease; NF-κB p65, nuclear factor kappa B p65; NLRP3, Nod-like receptor family pyrin domain containing 3; PD, Parkinson’s disease; QA, quinolinic acid; TLR4, Toll-like receptor 4; TNF-α, tumor necrosis factor alpha; 6-OHDA, 6-hydroxydopamine.

## Limitations

Phytochemicals are derived from various plant sources, and their composition can vary significantly depending on factors such as plant species, growing conditions, and processing methods. This variability may affect the consistency and comparability of results across studies. The response to phytochemicals and their effects on the GBA can vary among individuals due to genetic, environmental, and lifestyle factors. This interindividual variability should be considered when interpreting the potential therapeutic implications (Table 2).

**TABLE 2:** Clinical trials evidence

NCT number	Phytochemical	Found in	Condition/disorder	Dose	Sex of participants	Age of participants	*n*; country	Phase	Status	Results (if any)
NCT02415374	Avenanthramides (A,B,C)	Oats	Bioavailability; metabolism; Avenanthramides	229.6 mg/kg and 32.7 mg/kg	All	20–45 y	16; —	—	Completed	AVAs are absorbed in the plasma. AVA-B has the slowest elimination rate and longest half-life compared to AVA-A and AVA-C, while AVA-C demonstrated the lowest plasma concentrations.
NCT01651793	Phytochemicals	Theobroma cacao	Mental fatigue	70 mg caffeine, 179 mg theobromine, 499 mg flavanols, and 1 packet of Truvia sweetener	All	18–34 y	24; United States	—	Completed	—
NCT04421716	Curcumin and ursolic Acid	Apple peels and turmeric	Bioavailability of phytonutrients	2 wk + 3 d	All	≥18 y	18; United States	Early phase 1	Completed	—
NCT03213340	Catechin, curcuminoids, and flavonoid	Plants and turmeric	Biological aging	4 capsules catechin, 2 capsules curcuminoids, ∼1 oz. flavenoid)	All	≥65 y	39; United Kingdom	—	Completed	—
NCT01982734	Curcumin	Turmeric	Pharmacokinetics of new curcumin formulations	80 mg native powder + phytochemicals, micelles, or micelles + phytochemicals	All	≥18 y	23; Germany	Early phase 1	Completed	—
NCT02847117	Mastiha	Pistacia lentiscus	Biological availability	10 g	All	20–40 y	20; Greece	—	Completed	—
NCT03870126	Caffeine	Polyphenols	Mental energy and physical performance	75 mg	All	18–49 y	28; United States	—	Completed	—
NCT02561481	Sulforaphane	Cruciferous vegetables (e.g., broccoli, cauliflower, and broccoli sprouts)	ASD	1 μmol/lb (2.2 kg μmol/kg)	All	3–12 y	60; United States	Phase 2	Completed	Sulforaphane increased lipid peroxidation, and neuroinflammmation and reduced mitochondrial function and oxidative phosphorylation in ASD.
NCT01474993	Sulforaphane	Cruciferous vegetables such as broccoli, cauliflower, and broccoli sprouts	Autism	250 mg	All	13–30 y	44; United States	Phase 2	Completed	—
NCT01504854	Resveratrol	Red wine and the skin of red grapes	AD	500 mg	All	≥50 y	116; United States	Phase 2	Completed	Resveratrol decreases MMP9, CSF, induces adaptive immunity, and modulates neuroinflammation.
NCT02502253	Resveratrol	Red wine and the skin of red grapes	MCI	Low, moderate, high dose	All	50–90 y	14; United States	Phase 1	Completed	—
NCT02336633	Resveratrol	Knotweeds, pine trees, grape vines, raspberries, mulberries, peanut plants, cocoa bushes	HD	80 mg	All	≥18 y	102; France	—	Completed	—
NCT01699711	EGCG	Green tea	Down syndrome	9 mg/kg	All	14–29 y	87; Spain	Phase 2	Completed	EGCG improved visual recognition memory, inhibitory control, and adaptive behavior.
NCT00951834	EGCG	Green tea	AD	200–800 mg	All	≥60 y	21; Germany	Phase 2	Completed	—
NCT01699711	EGCG	Green tea	AD	9 mg/kg	All	14–29 y	87; Spain	Phase 2	Completed	Combining EGCG with cognitive training was more effective than just cognitive training or a placebo in improving visual recognition memory, inhibitory control, and adaptive behavior.
NCT00205179	Novasoy	Soybean	AD	100 mg	All	≥55 y	72; United States	Phase 2	Completed	Did not benefit cognition in older women and men with AD.
NCT01982578	Genistein	Lupin, fava beans, soybeans, kudzu, and psoralea	AD	60 mg	All	≥18 y	27; Spain	—	Completed	Genistein may to delay the onset of AD in prodromal AD patients with MCI.

Abbreviations: AD, Alzheimer’s disease; ASD, autism spectrum disorder; AVA, avenanthramides; CSF, cerebrospinal fluid; EGCG, epigallocatechin-3-gallate; HD, Huntington’s disease; MCI, mild cognitive impairment; MMP-9, matrix metallopeptidase 9.

The GBA is a complex network with multiple interacting components, including the gut microbiota, immune system, and neurotransmitter systems. Understanding the specific mechanisms and interactions involved in the effects of phytochemicals on this axis requires further research. Determining the optimal dosage, formulation, and delivery methods of phytochemicals for modulating the GBA is an ongoing challenge. Factors such as bioavailability, stability, and safety need to be considered when translating these findings into clinical applications. Furthermore, phytochemicals may interact with medications commonly used for neurological disorders. It is important to consider potential drug-phytochemical interactions and consult healthcare professionals when combining therapies.

## Conclusion

**FIGURE 2:**
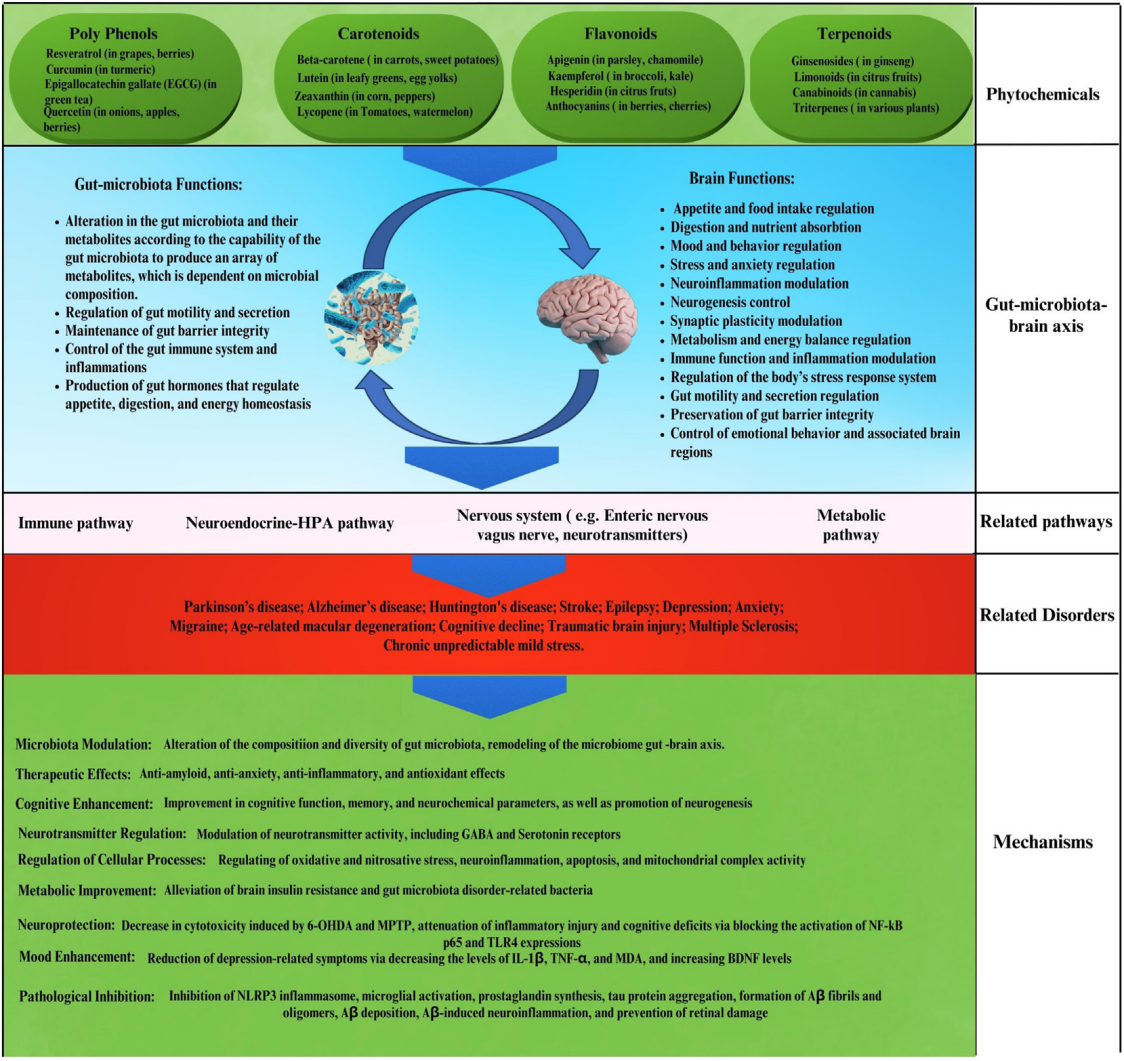
Classification of dietary phytochemicals.

In conclusion, this article highlights the potential therapeutic implications of phytochemicals on the GBA in neurological disorders (Figure 2). The GBA serves as a crucial communication network between the CNS and the ENS, and dysregulation of this axis has been associated with various neurological disorders. Phytochemicals, derived from plants, have shown promise in modulating the GBA through their interactions with the gut microbiota, immune system, and neurotransmitter systems. Compounds such as polyphenols, carotenoids, flavonoids, and terpenoids have been identified as having potential therapeutic benefits. Unlike many other nutrients, phytonutrients can directly reach the gut microbiota, exerting their influence without undergoing absorption processes.

By influencing the gut microbiota composition and function, phytochemicals may have the ability to impact brain function and potentially alleviate symptoms associated with neurological disorders such as PD, MS, depression, anxiety, and ASD. However, it is important to acknowledge that further research is needed to fully understand the optimal use of phytochemicals in neurological disorder treatment. Factors such as bioavailability, dosage, formulation, and potential interactions with other medications need to be carefully considered.

Overall, the findings suggest that phytochemicals have the potential to serve as therapeutic interventions for neurological disorders by modulating the GBA. Continued research in this area holds promise for developing novel approaches in the management and treatment of these complex conditions.

## Author contributions

The authors’ responsibilities were as follows – AT, AS: conceived the study; KRJ, VA, AT: wrote the initial draft; AS, PV, TJ, AS: reviewed and edited the original draft; and all authors: read and approved the final manuscript.

## Conflict of interest

The authors report no conflicts of interest.

## Funding

The authors reported no funding received for this study.

## Appendix A Supplementary data

Multimedia component1 Multimedia component1

## References

[1] Cryan J.F., O’Riordan K.J., Cowan C.S.M., Sandhu K.V., Bastiaanssen T.F.S., Boehme M. (2019). The microbiota-gut-brain axis. Physiol. Rev..

[2] Ullah H., Arbab S., Tian Y., Liu C.Q., Chen Y., Qijie L. (2023). The gut microbiota–brain axis in neurological disorder. Front. Neurosci..

[3] Wang J., Song Y., Chen Z., Leng S.X. (2018). Connection between systemic inflammation and neuroinflammation underlies neuroprotective mechanism of several phytochemicals in neurodegenerative diseases. Oxid. Med. Cell. Longev..

[4] Ahmadi A., Jamialahmadi T., Sahebkar A. (2022). Polyphenols and atherosclerosis: a critical review of clinical effects on LDL oxidation. Pharmacol. Res..

[5] Hosseini S.A., Zahedipour F., Sathyapalan T., Jamialahmadi T., Sahebkar A. (2021). Pulmonary fibrosis: therapeutic and mechanistic insights into the role of phytochemicals. Biofactors.

[6] Asgary S., Kelishadi R., Rafieian-Kopaei M., Najafi S., Najafi M., Sahebkar A. (2013). Investigation of the lipid-modifying and antiinflammatory effects of Cornus mas L. supplementation on dyslipidemic children and adolescents. Pediatr. Cardiol..

[7] Iranshahi M., Askari M., Sahebkar A., Hadjipavlou-Litina D. (2009). Evaluation of antioxidant, anti-inflammatory and lipoxygenase inhibitory activities of the prenylated coumarin umbelliprenin. Daru.

[8] Iranshahi M., Sahebkar A., Hosseini S.T., Takasaki M., Konoshima T., Tokuda H. (2010). Cancer chemopreventive activity of diversin from Ferula diversivittata in vitro and in vivo. Phytomedicine.

[9] Iranshahi M., Sahebkar A., Takasaki M., Konoshima T., Tokuda H. (2009). Cancer chemopreventive activity of the prenylated coumarin, umbelliprenin, in vivo. Eur. J. Cancer Prev..

[10] Shafiee M., Arekhi S., Omranzadeh A., Sahebkar A. (2018). Saffron in the treatment of depression, anxiety and other mental disorders: current evidence and potential mechanisms of action. J. Affect. Disord..

[11] Yaribeygi H., Mohammadi M.T., Rezaee R., Sahebkar A. (2018). Crocin improves renal function by declining Nox-4, IL-18, and p53 expression levels in an experimental model of diabetic nephropathy. J. Cell. Biochem..

[12] Yaribeygi H., Mohammadi M.T., Sahebkar A. (2018). Crocin potentiates antioxidant defense system and improves oxidative damage in liver tissue in diabetic rats. Biomed. Pharmacother..

[13] Momtazi A.A., Banach M., Pirro M., Katsiki N., Sahebkar A. (2017). Regulation of PCSK9 by nutraceuticals. Pharmacol. Res..

[14] Panahi Y., Fazlolahzadeh O., Atkin S.L., Majeed M., Butler A.E., Johnston T.P. (2019). Evidence of curcumin and curcumin analogue effects in skin diseases: a narrative review. J. Cell. Physiol..

[15] Sadeghi S., Davoodvandi A., Pourhanifeh M.H., Sharifi N., ArefNezhad R., Sahebnasagh R. (2019). Anti-cancer effects of cinnamon: insights into its apoptosis effects. Eur. J. Med. Chem..

[16] Sahebkar A. (2014). Curcuminoids for the management of hypertriglyceridaemia. Nat. Rev. Cardiol..

[17] Aqsa, Ali S., Summer M., Yousaf S., Nazakat L., Noor S. (2024). Pharmacological and immunomodulatory modes of action of medically important phytochemicals against arthritis: a molecular insight. Mol. Biol. Rep..

[18] Russo G.L., Spagnuolo C., Russo M. (2024). Reassessing the role of phytochemicals in cancer chemoprevention. Biochem. Pharmacol..

[19] Oslovsky V.E., Savelieva E.M., Drenichev M.S., Romanov G.A., Mikhailov S.N. (2020). Distinct peculiarities of in planta synthesis of isoprenoid and aromatic cytokinins. Biomolecules.

[20] Pandiella-Alonso A., Díaz-Rodríguez E., Sanz E. (2020). Antitumoral properties of the nutritional supplement ocoxin oral solution: a comprehensive review. Nutrients.

[21] Williamson G., Clifford M.N. (2010). Colonic metabolites of berry polyphenols: the missing link to biological activity?. Br. J. Nutr..

[22] Cryan J.F., O’Riordan K.J., Sandhu K., Peterson V., Dinan T.G. (2020). The gut microbiome in neurological disorders. Lancet Neurol.

[23] Moss J.W.E., Williams J.O., Ramji D.P. (2018). Nutraceuticals as therapeutic agents for atherosclerosis. Biochim. Biophys. Acta Mol. Basis Dis..

[24] Luo J., Lin X., Bordiga M., Brennan C., Xu B. (2021). Manipulating effects of fruits and vegetables on gut microbiota – a critical review. Int. J. Food Sci. Technol..

[25] Hewlings S.J., Kalman D.S. (2017). Curcumin: a review of its effects on human health. Foods.

[26] Rana A., Samtiya M., Dhewa T., Mishra V., Aluko R.E. (2022). Health benefits of polyphenols: a concise review. J. Food Biochem..

[27] Kawabata K., Mukai R., Ishisaka A. (2015). Quercetin and related polyphenols: new insights and implications for their bioactivity and bioavailability. Food Funct.

[28] Ruotolo R., Minato I., La Vitola P., Artioli L., Curti C., Franceschi V. (2020). Flavonoid-derived human phenyl-γ-valerolactone metabolites selectively detoxify amyloid-β oligomers and prevent memory impairment in a mouse model of Alzheimer’s disease. Mol. Nutr. Food Res..

[29] Qiu J., Chen Y., Zhuo J., Zhang L., Liu J., Wang B. (2022). Urolithin A promotes mitophagy and suppresses NLRP3 inflammasome activation in lipopolysaccharide-induced BV2 microglial cells and MPTP-induced Parkinson’s disease model. Neuropharmacology.

[30] Brown A.G. (1991). Nerve Cells and Nervous Systems: An Introduction to Neuroscience.

[31] Carabotti M., Scirocco A., Maselli M.A., Severi C. (2015). The gut-brain axis: interactions between enteric microbiota, central and enteric nervous systems. Ann. Gastroenterol..

[32] Man A.W.C., Xia N., Daiber A., Li H. (2020). The roles of gut microbiota and circadian rhythm in the cardiovascular protective effects of polyphenols. Br. J. Pharmacol..

[33] Amiot M.J., Riva C., Vinet A. (2016). Effects of dietary polyphenols on metabolic syndrome features in humans: a systematic review. Obes. Rev..

[34] Sun W., Shahrajabian M.H. (2023). Therapeutic potential of phenolic compounds in medicinal plants-natural health products for human health. Molecules.

[35] Johnson S.L., Kirk R.D., DaSilva N.A., Ma H., Seeram N.P., Bertin M.J. (2019). Polyphenol microbial metabolites exhibit gut and blood⁻brain barrier permeability and protect murine microglia against LPS-induced inflammation. Metabolites.

[36] Reddy V.P., Aryal P., Robinson S., Rafiu R., Obrenovich M., Perry G. (2020). Polyphenols in Alzheimer’s disease and in the gut–brain axis. Microorganisms.

[37] Tomás-Barberán F.A., Selma M.V., Espín J.C. (2016). Interactions of gut microbiota with dietary polyphenols and consequences to human health. Curr. Opin. Clin. Nutr. Metab. Care.

[38] Duda-Chodak A., Tarko T., Satora P., Sroka P. (2015). Interaction of dietary compounds, especially polyphenols, with the intestinal microbiota: a review. Eur. J. Nutr..

[39] Keppler K., Humpf H.U. (2005). Metabolism of anthocyanins and their phenolic degradation products by the intestinal microflora. Bioorg. Med. Chem..

[40] Makarewicz M., Drożdż I., Tarko T., Duda-Chodak A. (2021). The interactions between polyphenols and microorganisms, especially gut microbiota. Antioxidants.

[41] Cicero A.F.G., Sahebkar A., Fogacci F., Bove M., Giovannini M., Borghi C. (2020). Effects of phytosomal curcumin on anthropometric parameters, insulin resistance, cortisolemia and non-alcoholic fatty liver disease indices: a double-blind, placebo-controlled clinical trial. Eur. J. Nutr..

[42] Keihanian F., Saeidinia A., Bagheri R.K., Johnston T.P., Sahebkar A. (2018). Curcumin, hemostasis, thrombosis, and coagulation. J. Cell. Physiol..

[43] Marjaneh R.M., Rahmani F., Hassanian S.M., Rezaei N., Hashemzehi M., Bahrami A. (2018). Phytosomal curcumin inhibits tumor growth in colitis-associated colorectal cancer. J. Cell. Physiol..

[44] Mohajeri M., Sahebkar A. (2018). Protective effects of curcumin against doxorubicin-induced toxicity and resistance: a review. Crit. Rev. Oncol. Hematol..

[45] Mohammadi A., Blesso C.N., Barreto G.E., Banach M., Majeed M., Sahebkar A. (2019). Macrophage plasticity, polarization and function in response to curcumin, a diet-derived polyphenol, as an immunomodulatory agent. J. Nutr. Biochem..

[46] Mokhtari-Zaer A., Marefati N., Atkin S.L., Butler A.E., Sahebkar A. (2018). The protective role of curcumin in myocardial ischemia–reperfusion injury. J. Cell. Physiol..

[47] Kahkhaie K.R., Mirhosseini A., Aliabadi A., Mohammadi A., Mousavi M.J., Haftcheshmeh S.M. (2019). Curcumin: a modulator of inflammatory signaling pathways in the immune system. Inflammopharmacology.

[48] Rezaee R., Momtazi A.A., Monemi A., Sahebkar A. (2017). Curcumin: a potentially powerful tool to reverse cisplatin-induced toxicity. Pharmacol. Res..

[49] Ayati Z., Ramezani M., Amiri M.S., Moghadam A.T., Rahimi H., Abdollahzade A. (2019). Ethnobotany, phytochemistry and traditional uses of Curcuma spp. and pharmacological profile of two important species (C. longa and C. zedoaria): a review. Curr. Pharm. Des..

[50] Bagheri H., Ghasemi F., Barreto G.E., Rafiee R., Sathyapalan T., Sahebkar A. (2020). Effects of curcumin on mitochondria in neurodegenerative diseases. Biofactors.

[51] Aggarwal B.B., Sung B. (2009). Pharmacological basis for the role of curcumin in chronic diseases: an age-old spice with modern targets. Trends Pharmacol. Sci..

[52] Di Meo F., Margarucci S., Galderisi U., Crispi S., Peluso G. (2019). Curcumin, gut microbiota, and neuroprotection. Nutrients.

[53] Jazayeri S.D., Mustafa S., Manap M.Y., Ali A.M., Ismail A., Faujan N.H. (2009). Survival of bifidobacteria and other selected intestinal bacteria in TPY medium supplemented with curcumin as assessed in vitro. Int. J. Probiotics Prebiotics.

[54] Lou Y., Zheng J., Hu H., Lee J., Zeng S. (2015). Application of ultra-performance liquid chromatography coupled with quadrupole time-of-flight mass spectrometry to identify curcumin metabolites produced by human intestinal bacteria. J. Chromatogr. B Analyt. Technol. Biomed. Life. Sci..

[55] Rajeswari A., Sabesan M. (2008). Inhibition of monoamine oxidase-B by the polyphenolic compound, curcumin and its metabolite tetrahydrocurcumin, in a model of Parkinson’s disease induced by MPTP neurodegeneration in mice. Inflammopharmacology.

[56] Gao Y., Zhuang Z., Gao S., Li X., Zhang Z., Ye Z. (2017). Tetrahydrocurcumin reduces oxidative stress-induced apoptosis via the mitochondrial apoptotic pathway by modulating autophagy in rats after traumatic brain injury. Am. J. Transl. Res..

[57] Sun Z.Z., Li X.Y., Wang S., Shen L., Ji H.F. (2020). Bidirectional interactions between curcumin and gut microbiota in transgenic mice with Alzheimer’s disease. Appl. Microbiol. Biotechnol..

[58] Mena P., Bresciani L., Brindani N., Ludwig I.A., Pereira-Caro G., Angelino D. (2019). Phenyl-γ-valerolactones and phenylvaleric acids, the main colonic metabolites of flavan-3-ols: synthesis, analysis, bioavailability, and bioactivity. Nat. Prod. Rep..

[59] Carregosa D., Carecho R., Figueira I., Santos C.N. (2020). Low-molecular weight metabolites from polyphenols as effectors for attenuating neuroinflammation. J. Agric. Food Chem..

[60] Angelino D., Carregosa D., Domenech-Coca C., Savi M., Figueira I., Brindani N. (2019). 5-(hydroxyphenyl)-γ-valerolactone-sulfate, a key microbial metabolite of flavan-3-ols, is able to reach the brain: evidence from different in silico, in vitro and in vivo experimental models. Nutrients.

[61] Banc R., Rusu M.E., Filip L., Popa D.S. (2023). The impact of ellagitannins and their metabolites through gut microbiome on the gut health and brain wellness within the gut–brain axis. Foods.

[62] Garcia G., Pais T.F., Pinto P., Dobson G., McDougall G.J., Stewart D. (2020). Bioaccessible raspberry extracts enriched in ellagitannins and ellagic acid derivatives have anti-neuroinflammatory properties. Antioxidants.

[63] Villalba K.J.O., Barka F.V., Pasos C.V., Rodríguez P.E., Aires A. (2019). Tannins - Structural Properties, Biological Properties and Current Knowledge.

[64] Xu J., Yuan C., Wang G., Luo J., Ma H., Xu L. (2018). Urolithins attenuate LPS-induced neuroinflammation in BV2Microglia via MAPK, Akt, and NF-κB signaling pathways. J. Agric. Food Chem..

[65] Song X., Zhou B., Zhang P., Lei D., Wang Y., Yao G. (2016). Protective effect of silibinin on learning and memory impairment in LPS-treated rats via ROS–BDNF–TrkB pathway. Neurochem. Res..

[66] Keszthelyi D., Troost F.J., Masclee A.A.M. (2009). Understanding the role of tryptophan and serotonin metabolism in gastrointestinal function. Neurogastroenterol. Motil..

[67] O’Mahony S.M., Clarke G., Borre Y.E., Dinan T.G., Cryan J.F. (2015). Serotonin, tryptophan metabolism and the brain-gut-microbiome axis. Behav. Brain Res..

[68] Jenkins T.A., Nguyen J.C., Polglaze K.E., Bertrand P.P. (2016). Influence of tryptophan and serotonin on mood and cognition with a possible role of the gut-brain axis. Nutrients.

[69] Roth W., Zadeh K., Vekariya R., Ge Y., Mohamadzadeh M. (2021). Tryptophan metabolism and gut-brain homeostasis. Int. J. Mol. Sci..

[70] Jaeger B.N., Parylak S.L., Gage F.H. (2018). Mechanisms of dietary flavonoid action in neuronal function and neuroinflammation. Mol. Aspects Med..

[71] Lee Y., Jeon S.J., Lee H.E., Jung I.H., Jo Y.W., Lee S. (2016). Spinosin, a C-glycoside flavonoid, enhances cognitive performance and adult hippocampal neurogenesis in mice. Pharmacol. Biochem. Behav..

[72] Wang H., Zhao T., Liu Z., Danzengquzhen Cisangzhuoma, Ma J. (2023). The neuromodulatory effects of flavonoids and gut microbiota through the gut-brain axis. Front. Cell. Infect. Microbiol..

[73] Dey P. (2019). Gut microbiota in phytopharmacology: a comprehensive overview of concepts, reciprocal interactions, biotransformations and mode of actions. Pharmacol. Res..

[74] Josiah S.S., Famusiwa C.D., Crown O.O., Lawal A.O., Olaleye M.T., Akindahunsi A.A. (2022). Neuroprotective effects of catechin and quercetin in experimental Parkinsonism through modulation of dopamine metabolism and expression of IL-1β, TNF-α, NF-κB, IκKB, and p53 genes in male Wistar rats. Neurotoxicology.

[75] Xiong H.H., Lin S.Y., Chen L.L., Ouyang K.H., Wang W.J. (2023). The interaction between flavonoids and intestinal microbes: a review. Foods.

[76] Xie Y., Yang W., Tang F., Chen X., Ren L. (2015). Antibacterial activities of flavonoids: structure-activity relationship and mechanism. Curr. Med. Chem..

[77] Jameson K.G., Olson C.A., Kazmi S.A., Hsiao E.Y. (2020). Toward understanding microbiome-neuronal signaling. Mol. Cell.

[78] Devi L., Ohno M. (2012). 7,8-dihydroxyflavone, a small-molecule TrkB agonist, reverses memory deficits and BACE1 elevation in a mouse model of Alzheimer’s disease. Neuropsychopharmacology.

[79] Bollen E., Vanmierlo T., Akkerman S., Wouters C., Steinbusch H.M.W., Prickaerts J. (2013). 7,8-Dihydroxyflavone improves memory consolidation processes in rats and mice. Behav. Brain Res..

[80] Cowansage K.K., LeDoux J.E., Monfils M.H. (2010). Brain-derived neurotrophic factor: a dynamic gatekeeper of neural plasticity. Curr. Mol. Pharmacol..

[81] Gao L., Zhang Y., Sterling K., Song W. (2022). Brain-derived neurotrophic factor in Alzheimer’s disease and its pharmaceutical potential. Transl. Neurodegener..

[82] Zhang Z., Liu X., Schroeder J.P., Chan C.B., Song M., Yu S.P. (2014). 7,8-dihydroxyflavone prevents synaptic loss and memory deficits in a mouse model of Alzheimer’s disease. Neuropsychopharmacology.

[83] Ali F., Rahul, Naz F., Jyoti S., Siddique Y.H. (2017). Health functionality of apigenin: a review. Int. J. Food Prop..

[84] Zhao L., Wang J.L., Liu R., Li X.X., Li J.F., Zhang L. (2013). Neuroprotective, anti-amyloidogenic and neurotrophic effects of apigenin in an Alzheimer’s disease mouse model. Molecules.

[85] Loke W.M., Proudfoot J.M., Stewart S., McKinley A.J., Needs P.W., Kroon P.A. (2008). Metabolic transformation has a profound effect on anti-inflammatory activity of flavonoids such as quercetin: lack of association between antioxidant and lipoxygenase inhibitory activity. Biochem. Pharmacol..

[86] Ren S., Suo Q., Du W., Pan H., Yang M., Wang R. (2010). [Quercetin permeability across blood-brain barrier and its effect on the viability of U251 cells]. Sichuan Da Xue Xue Bao Yi Xue Ban.

[87] Moreno L.C.G.E.I., Puerta E., Suárez-Santiago J.E., Santos-Magalhães N.S., Ramirez M.J., Irache J.M. (2017). Effect of the oral administration of nanoencapsulated quercetin on a mouse model of Alzheimer’s disease. Int. J. Pharm..

[88] Xu M., Huang H., Mo X., Zhu Y., Chen X., Li X. (2021). Quercetin-3-O-glucuronide alleviates cognitive deficit and toxicity in Aβ_1-42_ -induced AD-like mice and SH-SY5Y cells. Mol. Nutr. Food Res..

[89] Rodriguez-Castaño G.P., Dorris M.R., Liu X., Bolling B.W., Acosta-Gonzalez A., Rey F.E. (2019). *Bacteroides thetaiotaomicron* starch utilization promotes quercetin degradation and butyrate production by *Eubacterium ramulus*. Front. Microbiol..

[90] Liu J., Wang F., Liu S., Du J., Hu X., Xiong J. (2017). Sodium butyrate exerts protective effect against Parkinson's disease in mice via stimulation of glucagon like peptide-1. J. Neurol. Sci..

[91] Govindarajan N., Agis-Balboa R.C., Walter J., Sananbenesi F., Fischer A. (2011). Sodium butyrate improves memory function in an Alzheimer's disease mouse model when administered at an advanced stage of disease progression. J. Alzheimers Dis..

[92] Fernando W.M.A.D.B., Martins I.J., Morici M., Bharadwaj P., Rainey-Smith S.R., Lim W.L.F. (2020). Sodium butyrate reduces brain amyloid-β levels and improves cognitive memory performance in an Alzheimer’s disease transgenic mouse model at an early disease stage. J. Alzheimers Dis..

[93] Ali T., Kim M.J., Rehman S.U., Ahmad A., Kim M.O. (2017). Anthocyanin-loaded PEG-gold nanoparticles enhanced the neuroprotection of anthocyanins in an Aβ_1–42_ mouse model of Alzheimer’s disease. Mol. Neurobiol..

[94] Kim K.H., Kim M.A., Moon E., Kim S.Y., Choi S.Z., Son M.W. (2011). Furostanol saponins from the rhizomes of *Dioscorea japonica* and their effects on NGF induction. Bioorg. Med. Chem. Lett..

[95] Cho T., Ryu J.K., Taghibiglou C., Ge Y., Chan A.W., Liu L. (2013). Long-term potentiation promotes proliferation/survival and neuronal differentiation of neural stem/progenitor cells. PLoS One.

[96] Woo K.W., Kwon O.W., Kim S.Y., Choi S.Z., Son M.W., Kim K.H. (2014). Phenolic derivatives from the rhizomes of *Dioscorea nipponica* and their anti-neuroinflammatory and neuroprotective activities. J. Ethnopharmacol..

[97] Reichardt L.F. (2006). Neurotrophin-regulated signalling pathways. Philos. Trans. R. Soc. Lond. B Biol. Sci..

[98] Nan S., Wang P., Zhang Y., Fan J. (2021). Epigallocatechin-3-gallate provides protection against Alzheimer’s disease-induced learning and memory impairments in rats. Drug Des. Devel. Ther..

[99] Liu M., Chen F., Sha L., Wang S., Tao L., Yao L. (2014). (-)-Epigallocatechin-3-gallate ameliorates learning and memory deficits by adjusting the balance of TrkA/p75NTR signaling in APP/PS1 transgenic mice. Mol. Neurobiol..

[100] Yang J., Song S., Li J., Liang T. (2014). Neuroprotective effect of curcumin on hippocampal injury in 6-OHDA-induced Parkinson’s disease rat. Pathol. Res. Pract..

[101] Carito V., Venditti A., Bianco A., Ceccanti M., Serrilli A.M., Chaldakov G. (2014). Effects of olive leaf polyphenols on male mouse brain NGF, BDNF and their receptors TrkA, TrkB and p75. Nat. Prod. Res..

[102] Rehman M.U., Sehar N., Dar N.J., Khan A., Arafah A., Rashid S. (2023). Mitochondrial dysfunctions, oxidative stress and neuroinflammation as therapeutic targets for neurodegenerative diseases: an update on current advances and impediments. Neurosci. Biobehav. Rev..

[103] Wang J., Song Y., Gao M., Bai X., Chen Z. (2016). Neuroprotective effect of several phytochemicals and its potential application in the prevention of neurodegenerative diseases. Geriatrics (Basel).

[104] Liu C.B., Wang R., Yi Y.F., Gao Z., Chen Y.Z. (2018). Lycopene mitigates β-amyloid induced inflammatory response and inhibits NF-κB signaling at the choroid plexus in early stages of Alzheimer’s disease rats. J. Nutr. Biochem..

[105] Heng Y., Zhang Q.S., Mu Z., Hu J.F., Yuan Y.H., Chen N.H. (2016). Ginsenoside Rg1 attenuates motor impairment and neuroinflammation in the MPTP-probenecid-induced parkinsonism mouse model by targeting α-synuclein abnormalities in the substantia nigra. Toxicol. Lett..

[106] de Sá Coutinho D., Pacheco M.T., Frozza R.L., Bernardi A. (2018). Anti-inflammatory effects of resveratrol: mechanistic insights. Int. J. Mol. Sci..

[107] Cho S., Namkoong K., Shin M., Park J., Yang E., Ihm J. (2017). Cardiovascular protective effects and clinical applications of resveratrol. J. Med. Food.

[108] Singh A.P., Singh R., Verma S.S., Rai V., Kaschula C.H., Maiti P. (2019). Health benefits of resveratrol: evidence from clinical studies. Med. Res. Rev..

[109] Omraninava M., Razi B., Aslani S., Imani D., Jamialahmadi T., Sahebkar A. (2021). Effect of resveratrol on inflammatory cytokines: a meta-analysis of randomized controlled trials. Eur. J. Pharmacol..

[110] Xiang S., Liu F., Lin J., Chen H., Huang C., Chen L. (2017). Fucoxanthin inhibits β-amyloid assembly and attenuates β-amyloid oligomer-induced cognitive impairments. J. Agric. Food Chem..

[111] Novelle M.G., Wahl D., Diéguez C., Bernier M., De Cabo R. (2015). Resveratrol supplementation: where are we now and where should we go?. Ageing Res. Rev..

[112] Dyck J.R.B., Schrauwen P. (2015). Resveratrol: challenges in translating pre-clinical findings to improved patient outcomes. Biochim. Biophys. Acta.

[113] Singh C.K., Ndiaye M.A., Ahmad N. (2015). Resveratrol and cancer: challenges for clinical translation. Biochim. Biophys. Acta.

[114] Sahebkar A. (2013). Effects of resveratrol supplementation on plasma lipids: a systematic review and meta-analysis of randomized controlled trials. Nutr. Rev.

[115] Sahebkar A., Serban C., Ursoniu S., Wong N.D., Muntner P., Graham I.M. (2015). Lack of efficacy of resveratrol on C-reactive protein and selected cardiovascular risk factors--results from a systematic review and meta-analysis of randomized controlled trials. Int. J. Cardiol..

[116] Buhrmann C., Popper B., Aggarwal B.B., Shakibaei M. (2017). Resveratrol downregulates inflammatory pathway activated by lymphotoxin α (TNF-β) in articular chondrocytes: comparison with TNF-α. PLoS One.

[117] Chung J.Y., Jeong J.H., Song J. (2020). Resveratrol modulates the gut-brain axis: focus on glucagon-like peptide-1, 5-HT, and gut microbiota. Front. Aging Neurosci..

[118] Yu Y.C., Li J., Zhang M., Pan J.C., Yu Y., Zhang J.B. (2019). Resveratrol improves brain-gut axis by regulation of 5-HT-dependent signaling in the rat model of irritable bowel syndrome. Front. Cell. Neurosci..

[119] Dou Z., Rong X., Zhao E., Zhang L., Lv Y. (2019). Neuroprotection of resveratrol against focal cerebral ischemia/reperfusion injury in mice through a mechanism targeting gut-brain axis. Cell. Mol. Neurobiol..

[120] Boyina H.K., Geethakhrishnan S.L., Panuganti S., Gangarapu K., Devarakonda K.P., Bakshi V. (2020). In silico and in vivo studies on quercetin as potential anti-Parkinson agent. Adv. Exp. Med. Biol..

[121] Balasubramanian R., Bazaz M.R., Pasam T., Sharief N., Velip L., Samanthula G. (2023). Involvement of microbiome gut–brain axis in neuroprotective effect of quercetin in mouse model of repeated mild traumatic brain injury. Neuromolecular Med.

[122] Chen T.J., Feng Y., Liu T., Wu T.T., Chen Y.J., Li X. (2020). Fisetin regulates gut microbiota and exerts neuroprotective effect on mouse model of Parkinson’s disease. Front. Neurosci..

[123] Cui C., Han Y., Li H., Yu H., Zhang B., Li G. (2022). Curcumin-driven reprogramming of the gut microbiota and metabolome ameliorates motor deficits and neuroinflammation in a mouse model of Parkinson’s disease. Front. Cell. Infect. Microbiol..

[124] Li C., Wang N., Zheng G., Yang L. (2021). Oral administration of resveratrol-selenium-peptide nanocomposites alleviates Alzheimer’s disease-like pathogenesis by inhibiting Aβ aggregation and regulating gut microbiota. ACS Appl. Mater. Interfaces.

[125] Johnson S.L., Park H.Y., Vattem D.A., Grammas P., Ma H., Seeram N.P. (2020). Equol, a blood–brain barrier permeable gut microbial metabolite of dietary isoflavone daidzein, exhibits neuroprotective effects against neurotoxins induced toxicity in human neuroblastoma SH-SY5Y cells and *Caenorhabditis elegans*. Plant Foods Hum. Nutr..

[126] Kosari-Nasab M., Shokouhi G., Ghorbanihaghjo A., Abbasi M.M., Salari A.A. (2018). Hesperidin attenuates depression-related symptoms in mice with mild traumatic brain injury. Life Sci.

[127] Liu Y.M., Shen J.D., Xu L.P., Li H.B., Li Y.C., Yi L.T. (2017). Ferulic acid inhibits neuro-inflammation in mice exposed to chronic unpredictable mild stress. Int. Immunopharmacol..

[128] Liaqat H., Parveen A., Kim S.Y. (2022). Neuroprotective natural products’ regulatory effects on depression via gut–brain axis targeting tryptophan. Nutrients.

[129] Esselun C., Theyssen E., Eckert G.P. (2021). Effects of urolithin A on mitochondrial parameters in a cellular model of early Alzheimer disease. Int. J. Mol. Sci..

[130] Fakhri S., Dargahi L., Abbaszadeh F., Jorjani M. (2018). Astaxanthin attenuates neuroinflammation contributed to the neuropathic pain and motor dysfunction following compression spinal cord injury. Brain Res. Bull..

[131] Sheikhpour E., Mard S.A., Farbood Y., Bavarsad K., Sarkaki A. (2023). The effects of gallic acid and vagotomy on motor function, intestinal transit, brain electrophysiology and oxidative stress alterations in a rat model of Parkinson’s disease induced by rotenone. Life Sci.

[132] Parsamanesh N., Asghari A., Sardari S., Tasbandi A., Jamialahmadi T., Xu S. (2021 Aug). Resveratrol and endothelial function: A literature review. Pharmacol Res.

