# Supplementary material for: Modulatory Effects of Phytochemicals on Gut–Brain Axis: Therapeutic Implication
Source: Curr Dev Nutr. 2024 May 22;8(6):103785. doi: 10.1016/j.cdnut.2024.103785 (PMC11208951; doi:10.1016/j.cdnut.2024.103785)
Supplement: Multimedia component1 [file mmc1.docx]

**Modulatory effects of phytochemicals on gut-brain axis: therapeutic implication**

Khojasteh Rahimi Jaberi et al.

***Gut-Brain Axis***

The control of several physiological processes, including food intake, immunological response, and sleep, is significantly influenced by the interconnections between the stomach and brain (1). Although it has been suggested for a long time that changes in these interactions are linked to chronic abdominal pain and gastrointestinal dysfunction, it is only recently that professional societies have started using the term "disorders of altered gut-brain interactions “instead of “ functional gastrointestinal disorders" (2, 3). In the neurological system the communication, between the brain and microbiota mostly happens through the vagus nerve and spinal cord; in the endocrine pathway, communication primarily occurs through the hypothalamic-pituitary-adrenal (HPA) axis (4). Cytokines are crucial in communication through the immune pathway. Furthermore, the interaction between the gut microbiota and the brain is influenced by metabolites from both the diet and gut microorganisms (4). The GBA is commonly linked to behaviors and neurotransmission (4). The GBA plays a crucial role in monitoring and integrating intestinal functions, and connects the brain's emotional and cognitive regions to the intestines. Disorders affecting the nervous system are often responsible for severe physical or psychological complications (5).

There are some vital and known mechanisms other pathways that are part of the brain-gut axis which describe in the following. Figure 1 illustrates Modulatory effects of phytochemicals on gut-brain axis. The ENS of the stomach is an intricate neuronal network that controls the gastrointestinal tract's functions. Via the vagus nerve and other neural connections, the ENS and the CNS exchange information. The ENS also contains enteroendocrine cells that produce gut hormones, such as cholecystokinin, ghrelin, and peptide YY (36-amino acid), which regulate appetite, digestion, and energy homeostasis (6). The gastrointestinal tract is home to a varied collection of microorganisms known as the gut microbiota. Through metabolic, immunological, endocrine, and neurological pathways, the gut microbiota communicates with the CNS. It generates metabolites that might affect the CNS and ENS, such as short-chain fatty acids (SCFAs). Additionally, it can modulate the immune system, which can affect the CNS (7). *Via* the ENS and the gut microbiota, it can affect the gut's operations, including motility, secretion, and immunological response. Through the production of gut hormones by enteroendocrine cells, the central CNS can also control appetite, food intake, and energy balance (6).

There is still debate regarding the precise contribution of peripheral (*e.g.* the gut) and central (such as the brain and spinal cord) mechanisms to the development of symptoms, despite tremendous advancements in research into the underlying causes of gut-brain disorders, such as functional dyspepsia and irritable bowel syndrome (IBS). Nonetheless, an increasing body of research indicates that dysregulation of signaling events involving the gut, enteric microbiota, ENS, and CNS is the source of persistent stomach discomfort. The immune system, gastrointestinal motility, visceral sensitivity, and mood are all impacted by this imbalance (2). In the past 15 years, the microbiome's research has produced a shift in our understanding of the GBA. We now see it as a complex system involving bidirectional interactions between the brain, gut, the ENS, the gut microbiota, and the immune system linked with the gut (8). This new understanding has also shed light on the etiology of neurological conditions that were once thought to be solely due to processes within the brain. This has opened up the possibility of using treatments that target the gut microbiota to address disorders of altered gut-brain interactions (formerly referred to as functional gastrointestinal diseases) in addition to neurological and mental conditions include ASD, PD, depression, anxiety, and Alzheimer's disease (AD) (8).

Much attention has been paid to the gut microbiota axis (GMA), which is a powerful but previously overlooked axis that facilitates communication between organs and the gut through a bidirectional communication network (9). Experimental studies exploring the microbiome, such as the impact of food on gut flora and its relationship with various diseases, have led to a re-evaluation of how communication occurs between the brain, liver, lung, kidney, joint, and stomach (10, 11). Researchers found that *Bacteroides fragilis* as a common component of the human colon bacteria can enhance defective intestine by straightforwardly focusing on tight junction expression, cytokine generation, and/or microbiome composition (12). In addition to new research suggesting connections between different organs and the gut microbiome, some studies also lend support to a holistic model that connects the gut microbiota with the neurological, cardiovascular, respiratory, and digestive systems (13, 14). Diet has a major impact on the gut microbiota's makeup, it then uses the GBA to affect the neurological system (15). One example is the role of tryptophan in regulating the homeostasis of the CNS after it is broken down into various bioactive compounds. These compounds act as GBA's chemical messengers, and are impacted by the GBA (16).

***Gut–Brain Axis under Physiological Conditions***

The intricate and interesting network connecting the intestinal and CNS is known as the GBA. This network is not only anatomical but also biochemical, as millions of nerves and neurons run between the gut and brain (17). Under physiological conditions, to maintain homeostasis, the GBA is essential in influencing various functions and behaviors (18). The GBA is significant in maintaining overall health and homeostasis that responsible for regulating appetite and food intake through the production of digestive hormones like ghrelin and these hormones send signals to the brain to stimulate or control energy homeostasis and reduce appetite (19). The gut microbiota can influence brain function through the production of neurotransmitters, and it can also modulate the immune system to regulate gut barrier function (19). Another role of GBA is modulation of mood and behavior *via* the gut microbiota's synthesis of neurotransmitters, including dopamine and serotonin (20). These neurotransmitters can affect the CNS and control emotional behavior and associated brain regions. The GBA is essential for preserving the integrity of the gut barrier, which prevents the entry of dangerous compounds, such pathogens, into the bloodstream. It can modulate the immune system and regulate the integrity of the gut barrier (21). Scientists have discovered that the tiny world of microbes living in our bodies can actually sway our actions by tinkering with the amygdala and hippocampus, two key brain areas. By comparing regular mice with germ-free ones, they noticed striking variations in the size and shape of these brain regions. The germ-free mice showed stunted nerve connections, fewer branches, and delicate spines, underscoring how crucial our gut bugs are in keeping our brain cells in tip-top shape (20). One of the GBA's key functions is regulation of gut motility and secretion through the ENS and the vagus nerve (**Figure 1**). The ENS can control gut motility and secretion independently of the CNS, but it can also communicate with the CNS to regulate these functions (17). The gut microbiota and the ENS allow the GBA to regulate the immune system. By generating metabolites that have an impact on immune cells, such SCFAs, the gut microbiota can control the immune system. The ENS can also regulate the immune system by producing neuropeptides that can modulate immune cell activity (22).

The GBA is a remarkable example of the intricate interplay between different organ systems, highlighting the importance of a holistic approach to health and wellness (17). The GBA is a fascinating area of research that has the potential to revolutionize our understanding of brain function and disease, as well as our approach to treating neurological disorders (18). It is a complex and dynamic system that is essential to the GBA, stress, and inflammation and allows the gut and brain to communicate with each other, influencing each other's functions and behaviors (18). This axis is a crucial component of the system of the body's stress reaction, with the HPA axis playing a key role in regulating this response and has implications for various physiological functions, including digestion, metabolism, and immune function (18).

The following physiological processes are mediated by the GBA: The GBA is essential for controlling digestion and the absorption of nutrients during digestion (23). The ENS, which is located in the intestinal wall, can communicate with the CNS through the neuroimmune and neuroendocrine signaling mediated by the vagus nerve (23). This communication allows the gut to respond to changes in food intake and regulate digestive processes accordingly (23). The GBA is involved in regulating metabolism and energy balance (18). Through the synthesis of SCFAs, the gut bacteria can affect metabolism, which can affect glucose and lipid metabolism. The regulation of hunger and fullness is another function of the GBA, which can affect food intake and energy balance (17). Another role of GBA is regulating immune function and inflammation. The gut microbiota can influence immune function through the production of metabolites and the regulation of immune cells (24). The HPA axis is crucial in controlling the body's stress response system, which is influenced by the GBA (24). The GBA also is involved in regulating mood and behavior through the production of neurotransmitters and metabolites (17). Gut microbiota has the ability to create dopamine and serotonin, among other neurotransmitters, which can affect mood, behavior, and cognition (17). It plays a role in regulating stress and anxiety, which can affect mood and behavior and involved in regulating brain function and plasticity (18). Brain function may be influenced by the gut bacteria through the production of metabolites and the regulation of neuroinflammation. Additionally, the GBA regulates neurogenesis and synaptic plasticity, two processes linked to memory and learning (17).

An intriguing illustration of how the body's many organ systems cooperate to preserve homeostasis and advance general health and wellbeing is the GBA. It is an intricate network that is essential to preserving homeostasis and regulating a range of physiological processes and behaviors. A vital component of the GBA, the gut microbiota is a complex colony of microbes that produce metabolites and neurotransmitters that have an impact on behavior and brain function. To properly comprehend the GBA and its possible therapeutic uses, more investigation is required.

***Microbiota–Brain–Gut Axis and Neurodegenerative Diseases/Brain Disorders***

Microbiota–Brain–Gut Axis is involved in the regulation of various brain disorders and neurodegenerative diseases (NDD) (19, 25). The Microbiota-brain-gut axis and neurodegenerative illnesses/brain disorders are discussed here. The Microbiota-brain-gut axis is a fascinating area of research that has the potential to revolutionize our understanding of brain function and disease, as well as our approach to treating neurological disorders (25). The diverse community of microorganisms is an important component of the microbiota-brain-gut axis, generating metabolites and neurotransmitters that have an impact on behavior and brain function (25). Numerous physiological aspects, including nutrition, stress, and inflammation, and has implications for various diseases, including neurodegenerative disorders. many neurodegenerative illnesses, including AD, PD, and Huntington's disease (HD), have been related to the gut-microbiota-brain- axis (25). Recognizing the function of the gut-brain-microbiota axis in these diseases could lead to novel therapeutic approaches. It has also been linked to various brain disorders, such as depression, anxiety, and autism spectrum disorder (25). Neurological illnesses have been related to the microbiota-brain-gut axis through regulation and involvement in numerous pathways, including neuroinflammation (26). Dysbiosis, an unbalanced makeup of the gut microbiota, can lead to immune activation and inflammation. Inflammation in the gut can trigger a systemic immune response and neuroinflammation, which are associated with NDD (26). Traumatic brain injury can induce acute bacterial dysbiosis within the fecal microbiome, leading to changes in gut microbiota composition that can contribute to NDD gut dysbiosis has been observed in patients with HD, and there is a correlation between the makeup of gut bacteria and, cognitive performance, and clinical outcomes (27). According to a meta-analysis, gut dysbiosis and PD are linked, and the gut microbiota can influence the immune system and the CNS by producing metabolites and neurotransmitters (27). MS patients have been shown to have dysbiosis in their guts, and through the synthesis of metabolites and neurotransmitters, the gut microbiota can influence the CNS and immune system (28). Alterations in the gut microbiota composition can affect neurotransmitter levels and contribute to NDD. On the other hand, certain beneficial gut bacteria can produce anti-inflammatory molecules and neuroprotective factors, which can help maintain brain health and protect against NDD (29).

Finding out how the microbiota, brain, and gut axis regulates neuroinflammation may help develop new treatment strategies (25). In a healthy host, the microbiota affects many physiological functions, including defense against pathogens, and has a significant role in behavior, brain development, and the host immune system (30). An imbalance in gut bacteria can influence neurological conditions. By altering the makeup of the gut microbiota, it paves the way for a surge of gut-bacteria-related substances and molecular patterns to migrate into lymphoid tissues in the gut. This movement, fueled by a compromised gut barrier and heightened intestinal permeability, plays a pivotal role in the emergence and advancement of a range of neurological disorders (30). The term "microbiota/GBA" refers to describe the bidirectional association between the microbiota and the brain. Thus, changes in the makeup of gut bacteria may have an impact on brain processes and vice versa (30). The gut microbiota has been found to have a significant impact on mental health, influencing mood, cognition, and behavior and it can produce molecules that influence mood, behavior, and cognition including dopamine and serotonin (30). It has been discovered to regulate a number of physiological processes by interacting with the brain *via* the microbiota-GBA (29). In summary, the mechanism of the microbiota-GBA in NDD involves the intricate interactions between gut microbiota, the CNS, and the immune system. Dysbiosis, immune activation, inflammation, neurotransmitter production, and communication in both directions between the brain and the stomach contribute to the pathogenesis of NDD. Understanding and modulating this axis has the capacity to create cutting-edge treatment plans for various illnesses.

***Overview of Phytochemicals***

Phytochemicals are natural bioactive compounds found in plant foods that have been associated with numerous health benefits (31). They found in a wide range of plant-based foods, including whole grains, vegetables, fruits, herbs, spices, teas, and red wine and they have been associated with numerous health benefits, including antioxidant, anti-inflammatory, and anti-cancer properties (31). Phytochemicals also help regulate blood sugar levels, lower cholesterol, and improve cardiovascular health (31). There are many different types of phytochemicals, including carotenoids, flavonoids, phenolic acids, and terpenoids. Each type of phytochemical has unique properties and health benefits (31). The bioavailability of phytochemicals can vary depending on the food source, processing, and cooking methods. For example, cooking can increase the bioavailability of certain phytochemicals, while others may be destroyed by heat (31). Phytochemicals have been used in herbalism and traditional medicine for centuries. Many medicinal plants contain phytochemicals that has been used to treat various ailments (31). While phytochemicals are similar to other nutrients found in plants, such as vitamins and minerals (32), there are some key differences. Phytochemicals are synthesized by plants in specific cell types, but are not essential to the plant's energy metabolism or anabolic or catabolic metabolism (32). In contrast, vitamins and minerals are essential nutrients that are required for the plant's growth and development. Phytochemicals have a diverse range of structures and chemical properties, which contribute to their unique health benefits (33) while vitamins and minerals has specific chemical structures and functions. Phytochemicals have bioactive properties that can influence various physiological processes in the body, such as antioxidant, anti-inflammatory, and anti-cancer properties (33). In contrast, vitamins and minerals have specific functions in the body, such as regulating metabolism and supporting immune function (32). The effects of phytochemicals on health can vary depending on the dosage. Some phytochemicals may have health benefits at low doses, while others may have adverse health effects at high doses (32). In contrast, vitamins and minerals has recommended daily intake levels that are required for optimal health. The bioavailability of phytochemicals can vary depending on the food source, processing, and cooking methods (32). In contrast, vitamins and minerals has specific absorption and utilization pathways in the body (32). Phytochemicals have been found to have a neuroprotective role in preventing NDD (34). These natural bioactive compounds found in plant-based foods protect neurons by targeting oxidative stress, mitochondrial dysfunction, neurotrophic factor deficit, apoptosis, and abnormal protein accumulation (34). Furthermore, they can also help regulate blood sugar levels, lower cholesterol, and improve cardiovascular health. They were found to have a neuroprotective role in preventing NDD, including AD and PD (35). These phytochemicals protect against neuronal damage and prevent the onset and progress of neurodegeneration by modifying pathogenic factors (35). Phytochemicals protect neurons by targeting multiple pathogenic factors of neurodegenerative disorders. They can also regulate neurotrophins, which are essential for the survival, development, and function of neurons (35). They have been proposed as a therapeutic strategy for age-dependent neurodegeneration (36). Intervention with food-derived compounds has been proposed as a therapeutic strategy for NDD (36). In conclusion, phytochemicals have a neuroprotective role in preventing NDD by protecting neurons and modifying pathogenic factors. They are found in a wide range of plant-based foods and have unique properties and health benefits. Phytochemicals have been proposed as a therapeutic strategy for age-dependent neurodegeneration, but more research is needed to establish their efficacy and safety. Understanding the role of phytochemicals in promoting brain health and preventing NDD could lead to novel therapeutic approaches.

***Classification of Phytochemicals***

Based on phytochemical’s function in plant metabolism, they are classified as primary and secondary metabolites (**Figure 1&2**).

Primary metabolites include amino acids, common sugars, proteins, purines, lipids, chlorophylls and pyrimidines of nucleic acids, which are necessary for plant life. Unlike the primary metabolites, secondary metabolites, such as terpenes, lignans, alkaloids, phenolics, flavonoids, plant steroids, saponins, curcumin and glucosides are remaining plant chemicals that are not necessary for plant life and produced by the cells through metabolic pathways (37, 38). Secondary metabolites are responsible for protecting plants from pathogens due to their antibiotic, antiviral and antifungal properties (39). Medicinal effects of the plants come from secondary metabolites and these molecules have been utilized for centuries in traditional medicine which associated with valuable industries, such as pharmaceutics, cosmetics and fine chemicals (40, 41). Based on their biosynthetic pathway of secondary metabolites, they are classified into four main groups: (a) terpenes (b) nitrogen-containing compounds, such as glucosinolates, cyanogenic glycosides and alkaloids (c) phenolic compounds, such as flavonoids and phenylpropanoids, (d) organosulfur compounds (40).

***4.1 Phenolic (polyphenol) compounds***

Phenolic compounds represent the largest category of phytochemicals, commonly present in the plant kingdom and have one or more hydroxyl groups which derived from phenylpropanoid, pentose phosphate and shikimate pathways (42). Besed on phenolics basic skeleton, different classes of phenolics have been categorized, such as C6 (simple phenol, benzoquinones), C6—C2 (acetophenone, phenylacetic acid), C6—C3 (hydroxycinnamic acid, coumarin, phenylpropanes, chromones), C6—C4 (naphthoquinones), C6—C1—C6 (xanthones), C6—C2—C6 (stilbenes, anthraquinones), and C6—C3—C6 (flavonoids, isoflavonoids, neoflavonoids) (43-46). Flavonoids, phenolicacids and polyphenols are the most important groups of dietary phenolics.

Flavonoids are the largest group of plant phenols that are ubiquitous in nature and occur as glucosides, methylated and aglycones derivatives (47). More than 4,000 flavonoids with favourable biochemical and antioxidant effects have been found in several parts of the fruits, vegetables and certain beverages (48). In recent years, because of broad biological and pharmacological activities of flavonoids, including, anti-microbial, cytotoxic, anti-inflammatory and powerful antioxidants effects, they have gained a lot of attention and many studies evaluate antioxidant effects associated with various diseases, such as cancer, AD, and atherosclerosis (14, 49).

Resveratrol, a natural polyphenol found in various foods and traditional Chinese medicines, has shown potential in treating NDD. Its molecular mechanisms of action have not been thoroughly studied, but pharmacokinetics and network pharmacology approaches have identified its main properties and potential targets. Through protein-protein interaction network analysis, AKT Serine/Threonine Kinase 1 (AKT1), tumor protein P53 (TP53), interleukin-6 (IL6), caspase 3 (CASP3), vascular endothelial growth factor A (VEGFA), tumor necrosis factor (TNF), MYC, mitogen-activated protein kinase (MAPK) 3, MAPK8, and albumin were identified as hub target genes with strong affinity for resveratrol. Experimental results showed that resveratrol increased Bcl-2 levels and reduced Bax and Caspase-3 expression in hippocampal neurons exposed to glutamate which function as a potential neuroprotective agent and attenuate the deleterious effects of focal cerebral ischemia/reperfusion-induced brain injury (50).

Curcumin as a phenolic compound has potential benefits in NDD, such as AD, and HD. It exhibits anti-inflammatory. Antioxidant and anti-amyloid properties, inhibiting the formation of beta-amyloid plaques and reducing neuroinflammation. Curcumin also has been shown to enhance brain-derived neurotrophic factor (BDNF) levels. BDNF is a protein that plays a crucial role in the growth, survival, and maintenance of neurons. Low levels of BDNF have been associated with NDD, and curcumin's ability to increase BDNF levels may contribute to its potential neuroprotective effects (51).

Phenolic acids and their derivatives (hydroxybenzoic acids and hydroxycinnamic acids) distributed widely in vegetables, fruits and cereal grains (52). Hydroxycinnamic acids possess phenylpropanoid C6-C3 structure, while hydroxybenzoic acids have general C6-C1 skeletons (53). Hydroxycinnamic acids and hydroxycinnamic acids have been studied mainly in health because they are known to be potent antioxidants against oxidative damage leading to various degenerative diseases (54). More than 8,000 polyphenolic compounds with antioxidant properties have been identified in fruits, vegetables, cereals and beverages (55). Because of their beneficial effects on human health, such as defense against ultraviolet radiation or aggression by pathogens, polyphenols are the subject of increasing scientific interest (56). Due to antioxidant properties of polyphenols, meta-analyses study strongly suggested that long term consumption of polyphenols offered can inhibit development of diabetes, osteoporosis, cancers, cardiovascular and NDD (57, 58).

***Terpenes***

Terpenes (terpinene, pinene, myrcene, limonene, *p*-cymene) are defined by the 2-methylbuta-1,3-diene (isoprene) units with general chemical formula (C_5_H_8_)_n_ (59). All the terpenes have even numbers of intact isoprene units, and most of the terpenoids have multi cyclic structures that differ from one another (60). By now, around 2,000 monoterpenes, sesquiterpenes and diterpenes have been described and many of these terpenes are use as flavours and fragrances in cosmetics and foods (61, 62). Terpenes are the largest group of naturally occurring secondary metabolites and can be found in plants and trees, such as thyme, tea, citrus fruits like lemon or mandarin and cannabis which widely used in aroma and flavor additives, nutraceutical, food and beverage products, cosmetics, pharmaceutical, biofuel industry, perfumes, synthetic chemicals and rubber products (63).

Terpenes have different properties including, anticonvulsant activities, anti-aggregatory, anti-coagulation, sedative and analgesic, anticancer, antioxidant and anti-allergic which are essential in supporting human health and has a significant role to treat various types of diseases (64-67). Furthermore, several studies have demonstrated that these molecules have antimicrobial activities against both the antibiotic-resistant and antibiotic-susceptible bacteria through their abilities to inhibition of protein and DNA synthesis and promote cell rupture (68, 69). Recent decades have shown that certain terpenes, such as Borneol, bakuchiol, (+)-α-terpineol, (−)-β-pinene, (+)-α-pinene and Limonene could reduce inflammation symptoms by inhibiting multiple pathological steps in the inflammatory process (70-73). Terpens alleviate inflammation symptoms *via* decreasing the release of pro-inflammatory cytokines (IL-1β, IL-6, and TNF-α), upregulating of endogenous antioxidants, such as glutathione (GSH), superoxide dismutases (SODs) and catalase (CAT) (71), reduce the expression of genes associated with inflammation (IL-4 and IL-13) (70), downregulating the expression of NF-κB and p65 and increase the expression of SOD and activation of nuclear factors E2-related factor 2 (Nrf2) (73).

Ginsenosides, a group of triterpenoid saponins, can affect various signaling pathways related to oxidative stress, inflammation synaptic plasticity, apoptosis and neurogenesis. These pathways include PI3K/Akt, CREB/BDNF, Keap1/Nrf2, and NF-κB/NLRP3 inflammasome pathways (74). Ginsenoside Rg1 has various benefits in AD models. It reduces the accumulation of amyloid-β (Aβ1)-42 and p-Tau, activates the BDNF-TrkB pathway, improves hippocampal potential and memory, increases the expression of synaptic plasticity-related proteins, and improves behavior in elderly mice (75).

***Nitrogen-containing compounds***

Nitrogen forms many organic compounds including amines, amides, amino acids, imines, amino alcohols, diazonium salts, lactams, hydrazides, oximes, nitriles, nitro compounds, carbazides and heterocyclic compounds, such as tetrazole, triazole and imidazole. Nitrogen-containing compounds are essential to many living organisms, including plants (76). Three important types of these compounds found in plants are alkaloids, glucosinolates, and cyanogenic glycosides (77). Alkaloids are a diverse group of nitrogen-containing compounds found in many plant species (78). Plants produce alkaloids as a form of defense against herbivores and pathogens (79). Examples of alkaloids include caffeine, nicotine, and morphine (80). They often have pharmacological effects on humans and animals, and some are used in medicine (81). Alkaloids can have a range of effects on humans and animals. Some are toxic and can cause harm when consumed in large amounts, while others have medicinal properties and can be used to treat various conditions (82). For example, the alkaloid ephedrine is used to treat asthma and nasal congestion, while the alkaloid vincristine is used to treat certain types of cancer (83, 84). In addition, alkaloids are particularly well known as anti-inflammatory, cardioprotective and anesthetics agents (85, 86). Based on Heinrich and Amirkia data, between 2014 and 2020, different alkaloids including, aconitine, ajmaline, atropine, berberine, boldine, caffeine, canescine, cathine, cinchonidine, cocaine, codeine, and colchicine used in marketed medicines, drugs, and clinical environments (87). Glucosinolates are natural compounds found in the Brassicales order of plants including [cauliflower](https://www.sciencedirect.com/topics/pharmacology-toxicology-and-pharmaceutical-science/cauliflower), broccoli, mustard, cabbage, rapeseed and horseradish (88, 89). Over 130 different glucosinolate species have been discovered so far, all of which are synthesized from amino acid precursors through enzymatic processes that determine their chemical and biological properties (90, 91). These compounds are responsible for the pungent taste and smell of mustard, horseradish, and wasabi (92). When hydrolyzed by myrosinase, they produce D-glucose and other products, such as isothiocyanates (89). The function of glucosinolates in plants is not yet fully understood, but they are believed to play a role in defense against herbivores and microbes (93, 94). Glucosinolates have been shown to have nematocidal, bacteriocidal, fungicidal and allelopathic properties (95, 96). Recent studies have found that glucosinolates have many benefits, including regulating inflammation, stress response, and antioxidant activities, as well as having direct antimicrobial properties (97, 98). Sulforaphane, sulfur- and nitrogen-containing phytochemicals, exhibits neuroprotective effects by targeting proteins, regulating metabolic pathways, and activating the [Nrf2/ antioxidant response element (ARE)] pathway. This activation leads to increased expression of phase II enzymes and inhibits the translocation of NF-κB, resulting in a decrease in pro-inflammatory mediators, cytokines, and oxidative markers associated with neuronal apoptosis (99). In addition, Sulforaphane protects against programmed cell death induced by oxidative stress from Aβ-fragments by modulating the Bax/Bcl-2 ratio and reducing ROS levels through the activation of the Nrf2/ARE pathway (100).

They are also being researched for their potential cancer chemo-protective properties, with isothiocyanates showing the best anti-carcinogenic activity (101, 102). However, feeding livestock high levels of glucosinolates can cause negative effects, such as reduced feed intake and growth, gastrointestinal irritation, goiter, anemia, and liver and kidney damage (97).

***Organosulfur compounds***

Organosulfur compounds (OSC) are a type of organic substances that have sulfur atoms in their structure (103). The primary origins of OSC are broccoli, garlic, cauliflower, onion, brussel sprouts, meat, eggs, cabbage and fish. Allicin, s-allyl cysteine, cysteine, sulforaphane, methionine, sulfonylureas and lipoic acid are some of the most frequently extracted OSC varieties from various plant sources (104). OSC have many biological and pharmacological effects on humans, such as antioxidant activity, anti-ageing, anti-parasitic, antiviral, anti-microbial, anti-platelet, immunomodulatory, fibrinolytic, anti-hypertensive, anti-hyperlipidemic, anti-atherosclerotic and activities (104-107).

In addition, OSC plays a crucial role in preventing various human pathological conditions, including chronic inflammation, by reducing inflammatory mediators, such as prostaglandin (PG)E_2_, nitric oxide (NO), IL-1β, IL-1, and downregulating the key regulating factor (NF-κB) during inflammation. Inflammation is linked with acute and chronic pathological conditions, and OSC has anti-inflammatory properties that occur in a dose-dependent manner. OSC also has indirect mechanisms and co-functions that provide protective roles as antioxidants, chemoprotection, and neuroprotection (108). These activities are beneficial in the treatment of various pathological conditions including cardiovascular diseases, diabetes, cancer and neurodegenerative disorders.

S-allylmercapto-N-acetylcysteine (ASSNAC) is an OSC that suitable for treatment of various neurodegenerative disease including AD, PD, MS, peripheral neuropathy, spinal cord injury, Lewy body disease, stroke and cerebral ischemia trough inhibition of formation of aldehyde-protein adducts, protein carbonylation, protein aggregation, and resulting neuroinflammation (109).

***Bioavailability of Phytochemicals***

In the human body, the bioavailability is defined as substances of phytochemicals obtained from ingested materials and then absorbed into the bloodstream for further delivery into designated organs and tissues for exerting biological function (110). Typically, bioavailability of phytochemicals is determined by evaluating the levels of phytochemicals and their metabolites in the blood following consumption (111). The bioactivity of a phytochemical is the extent to which it induces positive effects on human health or some biomarker of human health, such as a reduction in low-density lipoprotein levels or decrease in heart disease (112). The challenge of bioavailability of phytochemicals refers to the limited absorption and utilization of these beneficial compounds by the human body. Factors, such as poor solubility in water, interactions with other dietary components, food processing methods, metabolism by enzymes, and individual variations in gut microbiota contribute to this challenge. Strategies to enhance bioavailability include combining phytochemicals with absorption-enhancing components, using suitable food processing techniques, and promoting a healthy gut microbiota (113).

Dietary phytochemicals typically follow a path that involves being consumed, digested, and transported across the gastrointestinal epithelium before entering the circulatory system (110). Many phytochemicals are only minimally transferred across the intestinal mucosa, and those that do cross in significant amounts are quickly metabolized by phase II enzymes. These enzymes convert potentially toxic molecules into water-soluble conjugates (114). After undergoing metabolism in the gut mucosa, a significant portion of the products are secreted back into the gut lumen (115). These products may either be further metabolized by the gut microflora or excreted in the feces (115). If any unmetabolized compounds enter the circulation, they are often converted during their first pass through the liver (116). As a result, it is the modified forms that reach the target tissues, rather than the original compound found in the plant (114).

Terpenes (organic compounds based on the isoprene unit with the general formula (C5H8)n) are highly lipophilic, which means they can easily cross biological membranes by passive diffusion (116). However, their solubility in the aqueous phase of the gut lumen is low, which affects their bioavailability (117). One exception is d-limonene, a monoterpene found in citrus peel oils and juices, which has anticarcinogenic effects in rodent models of cancer. Although d-limonene is not present in high concentrations in plasma, its major metabolite, perillic acid, has been shown to be biologically active (118). In a study conducted by Crowell *et al.* human volunteers were given d-limonene (100 mg/kg body weight) in the form of orange oil incorporated into a food product (119). The researchers measured the plasma concentrations of perillic acid, d-limonene, limonene-1,2-diol and dihydroperillic acid. They found only small amounts of unmetabolized d-limonene in the plasma, but the average concentrations of perillic acid, dihydroperillic acid, and limonene-1,2-diol were 35, 33, and 16 micromolar, respectively (119).

Lectins are proteins with diverse structures and high molecular weight. They are found in plants and animals, with their main role being the specific binding to carbohydrates, particularly those found in cell membranes (120). This property allows lectins to participate in bio-recognition processes and cellular agglutination reactions (121). Some plant lectins have evolved as natural pesticides and can be toxic to humans (122). Lectins are resistant to digestion in the gut and have limited potential for intestinal absorption due to their large size (123). However, they have a strong tendency to interact with glycoconjugation sites on the intestinal surfaces, leading to various biological effects in the gastrointestinal tract (124). In animals, lectins can stimulate excessive growth of intestinal epithelial cells. The effects of lectins on the gut mucosa occur primarily when they are directly introduced into the digestive system (125). It is unlikely that lectins are absorbed and distributed to other organs through circulation. One possible route for lectins to reach sub-epithelial tissues in the gut is through uptake and translocation by intestinal M cells, which are involved in the immune response (126). However, it is unclear if this mechanism is biologically in humans consuming lectins from conventional food sources.

Anthocyanins are a group of phenolic compounds found in fruit juices, berries, and wine. Studies have shown that only a small fraction of anthocyanins is absorbed from most food sources, with absorption occurring rapidly in the stomach and upper intestine (127, 128). The average bioavailability of anthocyanins is reported to be less than 1%, with peak plasma concentrations in the 10-50 nmol/L range. Unmetabolized anthocyanin glycosides are often detected in blood and urine, but their abundance may have been underestimated in studies that did not account for the instability of anthocyanin glucuronides and sulphates in urine (129).

Flavonols, like anthocyanins, are present in plants as water-soluble glycosides and are commonly found in fruits and vegetables at lower concentrations (130). Studies have shown that the absorption of quercetin glucosides from food is more efficient than that of quercetin aglycone (131, 132). The absorption of rhamnoglucoside, rutin, is even less efficient (133). This suggests that intact glucosides are absorbed *via* the specialized glucose transport channels of small intestinal epithelial cells. Flavonol glucosides interact with glucose transporters, but mainly act as weak inhibitors of glucose absorption (133). Further research using *in vitro* systems, animal models, and human volunteers has established the metabolic fate of quercetin and other polyphenols in humans (134, 135). Most quercetin glycosides are hydrolyzed by the digestive enzyme lactase phlorizin hydrolase and absorbed as free quercetin (136). Only the metabolized flavonoids (e.g. glucuronides, sulphates) are found in the blood, and it is these compounds that must be studied to understand the physiological effects of flavonoids in the human body (114, 137). Quercetin is absorbed and excreted more slowly than anthocyanins, with prolonged dietary supplementation leading to plasma concentrations in the 1-2 μmol/L range (138). A large proportion of ingested polyphenols remains in the gut lumen and is broken down to simpler, more readily absorbable compounds by the gut microflora (139). Bacterial metabolism of polyphenols leads to a complex range of metabolites, including aldehydes and phenolic acids, which may exert local anti-inflammatory activity in the gut lumen and be important for maintaining mucosal homeostasis and health (140).

The bioavailability of a diverse range of organic nitrogen compounds has not been thoroughly characterized in a systematic manner. However, factors, such as chemical structure, solubility, pH, presence of other compounds, cooking methods, and individual gut microbiota can contribute to variations in the bioavailability of alkaloids, glucosinolates, and cyanogenic glycosides.

The bioavailability and pharmacokinetics of four active alkaloids found in Corydalis saxicola Bunting (Yanhuanglian), a component of traditional Chinese medicine were investigated. it is indicated that these alkaloids exhibit favorable oral bioavailability properties in rats, with less than 15% of the drugs being absorbed into systemic circulation following oral administration (141). Moerover, glucosinolates, a group of sulphur-containing glucosides found in brassica vegetables was also analysed. After ingestion, intact glucosinolates may be broken down in the small intestine or colon, leading to the absorption of isothiocyanates. Isothiocyanates have been shown to have potential health benefits, including inducing Phase II enzymes, increasing the metabolism and detoxification of chemical carcinogens, and potentially protecting against certain cancers (142). The bioavailability of cyanide after consuming foods containing high levels of cyanogenic glycosides has been examined. The bioavailability of cyanide varied depending on the food source, with different peak levels of cyanide observed in the body after consumption of foods, such as cassava, bitter apricot kernels, linseed, and persipan paste (143).

**References**

1. Osadchiy V, Martin CR, Mayer EA. The Gut–Brain Axis and the Microbiome: Mechanisms and Clinical Implications. Clinical Gastroenterology and Hepatology. 2019;17(2):322-32.

2. Mayer EA, Nance K, Chen S. The Gut–Brain Axis. Annual Review of Medicine. 2022;73(1):439-53.

3. Schwetz I, Bradesi S, Mayer EA. Current insights into the pathophysiology of irritable bowel syndrome. Current Gastroenterology Reports. 2003;5(4):331-6.

4. Bonaz B, Bazin T, Pellissier S. The vagus nerve at the interface of the microbiota-gut-brain axis. Frontiers in neuroscience. 2018;12:49.

5. Vu LT, Bowser R. Fluid-based biomarkers for amyotrophic lateral sclerosis. Neurotherapeutics. 2017;14:119-34.

6. Kuwahara A, Matsuda K, Kuwahara Y, Asano S, Inui T, Marunaka Y. Microbiota-gut-brain axis: enteroendocrine cells and the enteric nervous system form an interface between the microbiota and the central nervous system. Biomedical Research. 2020;41(5):199-216.

7. Ullah H, Arbab S, Tian Y, Liu C-q, Chen Y, Qijie L, et al. The gut microbiota–brain axis in neurological disorder. Frontiers in Neuroscience. 2023;17.

8. Mayer EA. Gut feelings: the emerging biology of gut–brain communication. Nature Reviews Neuroscience. 2011;12(8):453-66.

9. Sampson TR, Mazmanian SK. Control of brain development, function, and behavior by the microbiome. Cell host & microbe. 2015;17(5):565-76.

10. Hsiao EY, McBride SW, Hsien S, Sharon G, Hyde ER, McCue T, et al. Microbiota modulate behavioral and physiological abnormalities associated with neurodevelopmental disorders. Cell. 2013;155(7):1451-63.

11. Danneskiold-Samsøe NB, Dias de Freitas Queiroz Barros H, Santos R, Bicas JL, Cazarin CBB, Madsen L, et al. Interplay between food and gut microbiota in health and disease. Food Research International. 2019;115:23-31.

12. Hsiao Elaine Y, McBride Sara W, Hsien S, Sharon G, Hyde Embriette R, McCue T, et al. Microbiota Modulate Behavioral and Physiological Abnormalities Associated with Neurodevelopmental Disorders. Cell. 2013;155(7):1451-63.

13. Clemmensen C, Müller TD, Woods SC, Berthoud H-R, Seeley RJ, Tschöp MH. Gut-brain cross-talk in metabolic control. Cell. 2017;168(5):758-74.

14. Illiano P, Brambilla R, Parolini C. The mutual interplay of gut microbiota, diet and human disease. The FEBS Journal. 2020;287(5):833-55.

15. Ma N, He T, Johnston LJ, Ma X. Host–microbiome interactions: the aryl hydrocarbon receptor as a critical node in tryptophan metabolites to brain signaling. Gut microbes. 2020;11(5):1203-19.

16. Agus A, Clément K, Sokol H. Gut microbiota-derived metabolites as central regulators in metabolic disorders. Gut. 2021;70(6):1174-82.

17. Appleton J. The Gut-Brain Axis: Influence of Microbiota on Mood and Mental Health. Integr Med (Encinitas). 2018;17(4):28-32.

18. Cryan JF, O'Riordan KJ, Cowan CSM, Sandhu KV, Bastiaanssen TFS, Boehme M, et al. The Microbiota-Gut-Brain Axis. Physiological Reviews. 2019;99(4):1877-2013.

19. Suganya K, Koo B-S. Gut–Brain Axis: Role of Gut Microbiota on Neurological Disorders and How Probiotics/Prebiotics Beneficially Modulate Microbial and Immune Pathways to Improve Brain Functions. International Journal of Molecular Sciences. 2020;21(20):7551.

20. Mohajeri MH, La Fata G, Steinert RE, Weber P. Relationship between the gut microbiome and brain function. Nutrition reviews. 2018;76(7):481-96.

21. Margolis KG, Cryan JF, Mayer EA. The Microbiota-Gut-Brain Axis: From Motility to Mood. Gastroenterology. 2021;160(5):1486-501.

22. Martin CR, Osadchiy V, Kalani A, Mayer EA. The Brain-Gut-Microbiome Axis. Cell Mol Gastroenterol Hepatol. 2018;6(2):133-48.

23. Carloni S, Rescigno M. Unveiling the gut-brain axis: structural and functional analogies between the gut and the choroid plexus vascular and immune barriers. Semin Immunopathol. 2022;44(6):869-82.

24. Rutsch A, Kantsjö JB, Ronchi F. The Gut-Brain Axis: How Microbiota and Host Inflammasome Influence Brain Physiology and Pathology. Frontiers in Immunology. 2020;11.

25. Sasso JM, Ammar RM, Tenchov R, Lemmel S, Kelber O, Grieswelle M, et al. Gut Microbiome–Brain Alliance: A Landscape View into Mental and Gastrointestinal Health and Disorders. ACS Chemical Neuroscience. 2023;14(10):1717-63.

26. Murguiondo-Pérez R, Vidal Alcántar-Garibay Ó, Weintraub Ben-Zión E, Blancarte-Hernández E, Tejerina-Marion E, Cristina Loza-López E, et al. The influence of gut brain axis in neurodegenerative diseases: short review. Revista mexicana de neurociencia. 2022;23(5):177-82.

27. Bicknell B, Liebert A, Borody T, Herkes G, McLachlan C, Kiat H. Neurodegenerative and Neurodevelopmental Diseases and the Gut-Brain Axis: The Potential of Therapeutic Targeting of the Microbiome. International Journal of Molecular Sciences. 2023;24(11):9577.

28. Zhang H, Chen Y, Wang Z, Xie G, Liu M, Yuan B, et al. Implications of Gut Microbiota in Neurodegenerative Diseases. Frontiers in Immunology. 2022;13.

29. Quigley EMM. Microbiota-Brain-Gut Axis and Neurodegenerative Diseases. Curr Neurol Neurosci Rep. 2017;17(12):94.

30. Suganya K, Koo BS. Gut-Brain Axis: Role of Gut Microbiota on Neurological Disorders and How Probiotics/Prebiotics Beneficially Modulate Microbial and Immune Pathways to Improve Brain Functions. Int J Mol Sci. 2020;21(20).

31. Leitzmann C. Characteristics and Health Benefits of Phytochemicals. Forsch Komplementmed. 2016;23(2):69-74.

32. Morand C, Tomas-Barberan FA. Contribution of plant food bioactives in promoting health effects of plant foods: why look at interindividual variability? Eur J Nutr. 2019;58(Suppl 2):13-9.

33. Krueger ES, Griffin LE, Beales JL, Lloyd TS, Brown NJ, Elison WS, et al. Bioavailable Microbial Metabolites of Flavanols Demonstrate Highly Individualized Bioactivity on In Vitro β-Cell Functions Critical for Metabolic Health. Metabolites. 2023;13(7).

34. Kumar G. Neuroprotective mechanisms by phytochemicals in neurological disorders. Frontiers in Neuroscience. 2023;17:1149639.

35. Prakash R. Role of Phytoconstituents: Neuroprotective Approach. Pharmacological Benefits of Natural Agents: IGI Global; 2023. p. 69-84.

36. Kumar GP, Khanum F. Neuroprotective potential of phytochemicals. Pharmacognosy reviews. 2012;6(12):81.

37. Ramawat K, Dass S, Mathur M. The chemical diversity of bioactive molecules and therapeutic potential of medicinal plants. Herbal drugs: ethnomedicine to modern medicine. 2009:7-32.

38. Hahn NI. Are phytoestrogens nature's cure for what ails us? A look at the research. Journal of the American Dietetic Association. 1998;98(9):974-7.

39. Bourgaud F, Gravot A, Milesi S, Gontier E. Production of plant secondary metabolites: a historical perspective. Plant science. 2001;161(5):839-51.

40. Jamwal K, Bhattacharya S, Puri S. Plant growth regulator mediated consequences of secondary metabolites in medicinal plants. Journal of applied research on medicinal and aromatic plants. 2018;9:26-38.

41. Liu Z, Wang H, Xie J, Lv J, Zhang G, Hu L, et al. The roles of cruciferae glucosinolates in disease and pest resistance. Plants. 2021;10(6):1097.

42. Lattanzio V, Kroon PA, Quideau S, Treutter D. Plant phenolics—secondary metabolites with diverse functions. Recent advances in polyphenol research. 2009;1:1-35.

43. Haslam E. Plant polyphenols: vegetable tannins revisited: CUP Archive; 1989.

44. Whiting DA. Natural phenolic compounds 1900–2000: a bird's eye view of a century's chemistry. Natural Product Reports. 2001;18(6):583-606.

45. Jaganath IB, Crozier A. Dietary flavonoids and phenolic compounds. Plant phenolics and human health: biochemistry, nutrition, and pharmacology. 2010;1:1-50.

46. Andersen OM, Markham KR. Flavonoids: chemistry, biochemistry and applications: CRC press; 2005.

47. Dai J, Mumper RJ. Plant phenolics: extraction, analysis and their antioxidant and anticancer properties. Molecules. 2010;15(10):7313-52.

48. Burak M, Imen Y. Flavonoids and their antioxidant properties. Turkiye Klin Tip Bil Derg. 1999;19(1):296-304.

49. Lee YK, Yuk DY, Lee JW, Lee SY, Ha TY, Oh KW, et al. (−)-Epigallocatechin-3-gallate prevents lipopolysaccharide-induced elevation of beta-amyloid generation and memory deficiency. Brain research. 2009;1250:164-74.

50. Wang W, Wang S, Liu T, Ma Y, Huang S, Lei L, et al. Resveratrol: multi-targets mechanism on neurodegenerative diseases based on network pharmacology. Frontiers in Pharmacology. 2020;11:694.

51. Darvesh AS, Carroll RT, Bishayee A, Novotny NA, Geldenhuys WJ, Van der Schyf CJ. Curcumin and neurodegenerative diseases: a perspective. Expert opinion on investigational drugs. 2012;21(8):1123-40.

52. Herrmann K, Nagel CW. Occurrence and content of hydroxycinnamic and hydroxybenzoic acid compounds in foods. Critical reviews in food science & nutrition. 1989;28(4):315-47.

53. Teixeira J, Gaspar A, Garrido EM, Garrido J, Borges F. Hydroxycinnamic acid antioxidants: an electrochemical overview. BioMed research international. 2013;2013.

54. Mandal SM, Chakraborty D, Dey S. Phenolic acids act as signaling molecules in plant-microbe symbioses. Plant signaling & behavior. 2010;5(4):359-68.

55. Pandey KB, Rizvi SI. Plant polyphenols as dietary antioxidants in human health and disease. Oxidative medicine and cellular longevity. 2009;2:270-8.

56. Beckman CH. Phenolic-storing cells: keys to programmed cell death and periderm formation in wilt disease resistance and in general defence responses in plants? Physiological and molecular plant pathology. 2000;57(3):101-10.

57. Hollman P, Arts I. Polyphenols and disease risk in epidemiological studies. Am J Clin Nutr. 2005;81:317-25.

58. Graf BA, Milbury PE, Blumberg JB. Flavonols, flavones, flavanones, and human health: epidemiological evidence. Journal of medicinal food. 2005;8(3):281-90.

59. Perveen S, Al-Taweel A. Terpenes and terpenoids: BoD–Books on Demand; 2018.

60. Barton D, Meth-Cohn O. Comprehensive natural products chemistry: Newnes; 1999.

61. Brahmkshatriya PP, Brahmkshatriya PS. Terpenes: Chemistry, biological role, and therapeutic applications. Natural products: phytochemistry, botany and metabolism of alkaloids, phenolics and terpenes. 2013:2665-91.

62. Hamburger M, Hostettmann K. 7. Bioactivity in plants: the link between phytochemistry and medicine. Phytochemistry. 1991;30(12):3864-74.

63. Prado-Audelo D, Luisa M, Cortés H, Caballero-Florán IH, González-Torres M, Escutia-Guadarrama L, et al. Therapeutic applications of terpenes on inflammatory diseases. Frontiers in Pharmacology. 2021:2114.

64. Silva BI, Nascimento EA, Silva CJ, Silva TG, Aguiar JS. Anticancer activity of monoterpenes: A systematic review. Molecular Biology Reports. 2021;48:5775-85.

65. Dzoyem JP, Melong R, Tsamo AT, Tchinda AT, Kapche DG, Ngadjui BT, et al. Cytotoxicity, antimicrobial and antioxidant activity of eight compounds isolated from Entada abyssinica (Fabaceae). BMC research notes. 2017;10:1-6.

66. Querio G, Antoniotti S, Foglietta F, Bertea CM, Canaparo R, Gallo MP, et al. Chamazulene attenuates ROS levels in bovine aortic endothelial cells exposed to high glucose concentrations and hydrogen peroxide. Frontiers in physiology. 2018;9:246.

67. Xiong H-H, Lin S-Y, Chen L-L, Ouyang K-H, Wang W-J. The interaction between flavonoids and intestinal microbes: A review. Foods. 2023;12(2):320.

68. Álvarez-Martínez F, Barrajón-Catalán E, Herranz-López M, Micol V. Antibacterial plant compounds, extracts and essential oils: An updated review on their effects and putative mechanisms of action. Phytomedicine. 2021;90:153626.

69. Álvarez-Martínez FJ, Barrajón-Catalán E, Herranz-López M, Micol V. Antibacterial plant compounds, extracts and essential oils: An updated review on their effects and putative mechanisms of action. Phytomedicine. 2021;90:153626.

70. Yang J, Choi W-S, Kim K-J, Eom C-D, Park M-J. Investigation of active anti-inflammatory constituents of essential oil from Pinus koraiensis (Sieb. et Zucc.) wood in LPS-stimulated RBL-2H3 cells. Biomolecules. 2021;11(6):817.

71. Islam AUS, Hellman B, Nyberg F, Amir N, Jayaraj RL, Petroianu G, et al. Myrcene attenuates renal inflammation and oxidative stress in the adrenalectomized rat model. Molecules. 2020;25(19):4492.

72. Kumar A, Sawhney G, Nagar RK, Chauhan N, Gupta N, Kaul A, et al. Evaluation of the immunomodulatory and anti-inflammatory activity of Bakuchiol using RAW 264.7 macrophage cell lines and in animal models stimulated by lipopolysaccharide (LPS). International Immunopharmacology. 2021;91:107264.

73. Bansod S, Chilvery S, Saifi MA, Das TJ, Tag H, Godugu C. Borneol protects against cerulein‐induced oxidative stress and inflammation in acute pancreatitis mice model. Environmental Toxicology. 2021;36(4):530-9.

74. Feng H, Xue M, Deng H, Cheng S, Hu Y, Zhou C. Ginsenoside and its therapeutic potential for cognitive impairment. Biomolecules. 2022;12(9):1310.

75. Choi RJ, Roy A, Jung HJ, Ali MY, Min B-S, Park CH, et al. BACE1 molecular docking and anti-Alzheimer's disease activities of ginsenosides. Journal of ethnopharmacology. 2016;190:219-30.

76. Schmeltz I, Hoffmann D. Nitrogen-containing compounds in tobacco and tobacco smoke. Chemical Reviews. 1977;77(3):295-311.

77. Rabizadeh F, Mirian MS, Doosti R, Kiani-Anbouhi R, Eftekhari E. Phytochemical Classification of Medicinal Plants Used in the Treatment of Kidney Disease Based on Traditional Persian Medicine. Evidence-Based Complementary and Alternative Medicine. 2022;2022.

78. Ng YP, Or TCT, Ip NY. Plant alkaloids as drug leads for Alzheimer's disease. Neurochemistry international. 2015;89:260-70.

79. Joosten L, van Veen JA. Defensive properties of pyrrolizidine alkaloids against microorganisms. Phytochemistry Reviews. 2011;10:127-36.

80. Rajput A, Sharma R, Bharti R. Pharmacological activities and toxicities of alkaloids on human health. Materials Today: Proceedings. 2022;48:1407-15.

81. Bribi N. Pharmacological activity of alkaloids: a review. Asian journal of botany. 2018;1(1):1-6.

82. Matsuura HN, Fett-Neto AG. Plant alkaloids: main features, toxicity, and mechanisms of action. Plant toxins. 2015;2(7):1-15.

83. Kukula-Koch W, Widelski J. Alkaloids. Pharmacognosy: Elsevier; 2017. p. 163-98.

84. El-Sakka MA. Phytochemistry (3) alkaloids. Al Azhar University, Faculty of Pharmacy, Department of Pharmacognosy: Cairo, Egypt. 2010.

85. Tsuchiya H. Membrane interactions of phytochemicals as their molecular mechanism applicable to the discovery of drug leads from plants. Molecules. 2015;20(10):18923-66.

86. Ushie O, Ochepo E, Nkom P, Ago M, Gani J. Medicinal Uses of Nitrogen Group Containing Secondary Metabolites: A. Tropical Journal of Science and Technology. 2023;4(1):71-82.

87. Amirkia V, Heinrich M. Alkaloids as drug leads–A predictive structural and biodiversity-based analysis. Phytochemistry letters. 2014;10:xlviii-liii.

88. Prieto M, López CJ, Simal-Gandara J. Glucosinolates: Molecular structure, breakdown, genetic, bioavailability, properties and healthy and adverse effects. Advances in food and Nutrition Research. 2019;90:305-50.

89. Ishida M, Hara M, Fukino N, Kakizaki T, Morimitsu Y. Glucosinolate metabolism, functionality and breeding for the improvement of Brassicaceae vegetables. Breeding science. 2014;64(1):48-59.

90. Mithen RF, Dekker M, Verkerk R, Rabot S, Johnson IT. The nutritional significance, biosynthesis and bioavailability of glucosinolates in human foods. Journal of the Science of Food and Agriculture. 2000;80(7):967-84.

91. Baskar V, Gururani MA, Yu JW, Park SW. Engineering glucosinolates in plants: current knowledge and potential uses. Applied biochemistry and biotechnology. 2012;168:1694-717.

92. Bell L, Oloyede OO, Lignou S, Wagstaff C, Methven L. Taste and flavor perceptions of glucosinolates, isothiocyanates, and related compounds. Molecular Nutrition & Food Research. 2018;62(18):1700990.

93. Yan X, Chen S. Regulation of plant glucosinolate metabolism. Planta. 2007;226:1343-52.

94. Bennett RN, Wallsgrove RM. Secondary metabolites in plant defence mechanisms. New phytologist. 1994;127(4):617-33.

95. Fahey JW, Zalcmann AT, Talalay P. The chemical diversity and distribution of glucosinolates and isothiocyanates among plants. Phytochemistry. 2001;56(1):5-51.

96. Prakash D, Gupta C. Glucosinolates: The phytochemicals of nutraceutical importance. Journal of Complementary and Integrative Medicine. 2012;9(1).

97. Bischoff KL. Glucosinolates. Nutraceuticals: Elsevier; 2021. p. 903-9.

98. Bischoff K. Glucosinolates and organosulfur compounds. Nutraceuticals in Veterinary Medicine. 2019:113-9.

99. Jazwa A, Rojo AI, Innamorato NG, Hesse M, Fernández-Ruiz J, Cuadrado A. Pharmacological targeting of the transcription factor Nrf2 at the basal ganglia provides disease modifying therapy for experimental parkinsonism. Antioxidants & redox signaling. 2011;14(12):2347-60.

100. Lee C, Park GH, Lee S-R, Jang J-H. Attenuation of-amyloid-induced oxidative cell death by sulforaphane via activation of NF-E2-related factor 2. Oxidative medicine and cellular longevity. 2013;2013.

101. Connolly EL, Sim M, Travica N, Marx W, Beasy G, Lynch GS, et al. Glucosinolates from cruciferous vegetables and their potential role in chronic disease: Investigating the preclinical and clinical evidence. Frontiers in pharmacology. 2021;12:767975.

102. Das S, Tyagi AK, Kaur H. Cancer modulation by glucosinolates: A review. Current science. 2000:1665-71.

103. Mahomoodally MF, Nabee N, Baureek N. Organosulfur compounds (allyl sulfide, indoles). Antioxidants Effects in Health: Elsevier; 2022. p. 417-26.

104. Walag AMP, Ahmed O, Jeevanandam J, Akram M, Ephraim-Emmanuel BC, Egbuna C, et al. Health benefits of organosulfur compounds. Functional foods and nutraceuticals: bioactive components, formulations and innovations. 2020:445-72.

105. Chu C-C, Wu W-S, Shieh J-P, Chu H-L, Lee C-P, Duh P-D. The anti-inflammatory and vasodilating effects of three selected dietary organic sulfur compounds from Allium species. Journal of functional biomaterials. 2017;8(1):5.

106. Munir MT. Effect of garlic on the health and performance of broilers. Veterinaria. 2015;3(1):32-9.

107. Schafer G, H Kaschula C. The immunomodulation and anti-inflammatory effects of garlic organosulfur compounds in cancer chemoprevention. Anti-Cancer Agents in Medicinal Chemistry (Formerly Current Medicinal Chemistry-Anti-Cancer Agents). 2014;14(2):233-40.

108. Ruhee RT, Roberts LA, Ma S, Suzuki K. Organosulfur compounds: A review of their anti-inflammatory effects in human health. Frontiers in Nutrition. 2020;7:64.

109. Ott DM. Organosulfur compounds for the prevention and treatment of neurodegenerative diseases. Google Patents; 2018.

110. Epriliati I, Ginjom IR. Bioavailability of phytochemicals. Phytochemicals—A Global Perspective of Their Role in Nutrition and Health. 2012:401-28.

111. Hu Y, Lin Q, Zhao H, Li X, Sang S, McClements DJ, et al. Bioaccessibility and bioavailability of phytochemicals: Influencing factors, improvements, and evaluations. Food Hydrocolloids. 2022:108165.

112. Dillard CJ, German JB. Phytochemicals: nutraceuticals and human health. Journal of the Science of Food and Agriculture. 2000;80(12):1744-56.

113. Hu Y, Lin Q, Zhao H, Li X, Sang S, McClements DJ, et al. Bioaccessibility and bioavailability of phytochemicals: Influencing factors, improvements, and evaluations. Food Hydrocolloids. 2023;135:108165.

114. Tiwari BK, Brunton N, Brennan CS. Handbook of plant food phytochemicals: Wiley Online Library; 2015.

115. Wang M, Zhao H, Wen X, Ho CT, Li S. Citrus flavonoids and the intestinal barrier: Interactions and effects. Comprehensive Reviews in Food Science and Food Safety. 2021;20(1):225-51.

116. Johnson IT. Phytochemicals and health. Handbook of Plant Food Phytochemicals: sources, stability and extraction. 2013:49-67.

117. Ninkuu V, Zhang L, Yan J, Fu Z, Yang T, Zeng H. Biochemistry of terpenes and recent advances in plant protection. International Journal of Molecular Sciences. 2021;22(11):5710.

118. Miller JA, Thompson PA, Hakim IA, Chow H-HS, Thomson CA. d-Limonene: a bioactive food component from citrus and evidence for a potential role in breast cancer prevention and treatment. Oncology Reviews. 2011;5:31-42.

119. Crowell PL, Elson CE, Bailey HH, Elegbede A, Haag JD, Gould MN. Human metabolism of the experimental cancer therapeutic agent d-limonene. Cancer chemotherapy and pharmacology. 1994;35:31-7.

120. Santos AF, Da Silva M, Napoleão T, Paiva P, Correia MdS, Coelho L. Lectins: Function, structure, biological properties andpotential applications. 2014.

121. Vasconcelos IM, Oliveira JTA. Antinutritional properties of plant lectins. Toxicon. 2004;44(4):385-403.

122. Wink M. Mode of action and toxicology of plant toxins and poisonous plants. Mitt Julius Kühn-Inst. 2009;421:93-112.

123. He S, Simpson BK, Sun H, Ngadi MO, Ma Y, Huang T. Phaseolus vulgaris lectins: A systematic review of characteristics and health implications. Critical reviews in food science and nutrition. 2018;58(1):70-83.

124. Smart JD. Lectin-mediated drug delivery in the oral cavity. Advanced drug delivery reviews. 2004;56(4):481-9.

125. Linderoth A, Prykhod’ko O, Pierzynowski SG, Weström BR. Enterally but not parenterally administered Phaseolus vulgaris lectin induces growth and precocious maturation of the gut in suckling rats. Neonatology. 2006;89(1):60-8.

126. Lyu S-Y, Park W-B. Transport of mistletoe lectin by M cells in human intestinal follicle-associated epithelium (FAE) in vitro. Archives of pharmacal research. 2008;31:1613-21.

127. Manach C, Williamson G, Morand C, Scalbert A, Rémésy C. Bioavailability and bioefficacy of polyphenols in humans. I. Review of 97 bioavailability studies. The American journal of clinical nutrition. 2005;81(1):230S-42S.

128. Teixeira M, De Luca L, Faria A, Bordiga M, de Freitas V, Mateus N, et al. First Insights on the Bioaccessibility and Absorption of Anthocyanins from Edible Flowers: Wild Pansy, Cosmos, and Cornflower. Pharmaceuticals [Internet]. 2024; 17(2).

129. Felgines C, Talavera S, Gonthier M-P, Texier O, Scalbert A, Lamaison J-L, et al. Strawberry anthocyanins are recovered in urine as glucuro-and sulfoconjugates in humans. The Journal of nutrition. 2003;133(5):1296-301.

130. Kaur C, Kapoor HC. Antioxidants in fruits and vegetables–the millennium’s health. International journal of food science & technology. 2001;36(7):703-25.

131. Murota K, Terao J. Antioxidative flavonoid quercetin: implication of its intestinal absorption and metabolism. Archives of biochemistry and biophysics. 2003;417(1):12-7.

132. Bentz AB. A Review of quercetin: chemistry, antioxident properties, and bioavailability. Journal of young investigators. 2017.

133. Hollman PC, de Vries JH, van Leeuwen SD, Mengelers MJ, Katan MB. Absorption of dietary quercetin glycosides and quercetin in healthy ileostomy volunteers. The American journal of clinical nutrition. 1995;62(6):1276-82.

134. van Duynhoven J, Vaughan EE, Jacobs DM, A. Kemperman R, van Velzen EJ, Gross G, et al. Metabolic fate of polyphenols in the human superorganism. Proceedings of the national academy of sciences. 2011;108(supplement_1):4531-8.

135. Rechner AR, Kuhnle G, Bremner P, Hubbard GP, Moore KP, Rice-Evans CA. The metabolic fate of dietary polyphenols in humans. Free Radical Biology and Medicine. 2002;33(2):220-35.

136. Day AJ, Gee JM, DuPont MS, Johnson IT, Williamson G. Absorption of quercetin-3-glucoside and quercetin-4′-glucoside in the rat small intestine: the role of lactase phlorizin hydrolase and the sodium-dependent glucose transporter. Biochemical pharmacology. 2003;65(7):1199-206.

137. Prasain JK, Barnes S. Metabolism and bioavailability of flavonoids in chemoprevention: current analytical strategies and future prospectus. Molecular pharmaceutics. 2007;4(6):846-64.

138. Conquer J, Maiani G, Azzini E, Raguzzini A, Holub B. Supplementation with quercetin markedly increases plasma quercetin concentration without effect on selected risk factors for heart disease in healthy subjects. The Journal of nutrition. 1998;128(3):593-7.

139. Forester SC, Waterhouse AL. Metabolites are key to understanding health effects of wine polyphenolics. The Journal of nutrition. 2009;139(9):1824S-31S.

140. Larrosa M, Luceri C, Vivoli E, Pagliuca C, Lodovici M, Moneti G, et al. Polyphenol metabolites from colonic microbiota exert anti‐inflammatory activity on different inflammation models. Molecular nutrition & food research. 2009;53(8):1044-54.

141. Li HL, Zhang WD, Zhang C, Liu RH, Wang XW, Wang XL, et al. Bioavailabilty and pharmacokinetics of four active alkaloids of traditional Chinese medicine Yanhuanglian in rats following intravenous and oral administration. J Pharm Biomed Anal. 2006;41(4):1342-6.

142. Johnson IT. Glucosinolates in the human diet. Bioavailability and implications for health. Phytochemistry Reviews. 2002;1(2):183-8.

143. Abraham K, Buhrke T, Lampen A. Bioavailability of cyanide after consumption of a single meal of foods containing high levels of cyanogenic glycosides: a crossover study in humans. Arch Toxicol. 2016;90(3):559-74.
